# New Triterpenoids from *Lansium domesticum* Corr. cv *kokossan* and Their Cytotoxic Activity

**DOI:** 10.3390/molecules28052144

**Published:** 2023-02-24

**Authors:** Tri Mayanti, Zulfikar, Sarah Fawziah, Al Arofatus Naini, Rani Maharani, Kindi Farabi, Nurlelasari, Muhammad Yusuf, Desi Harneti, Dikdik Kurnia, Unang Supratman

**Affiliations:** 1 Department of Chemistry, Faculty of Mathematics and Natural Sciences, Universitas Padjadjaran, Jatinangor 45363, West Java, Indonesia; 2 Central Laboratory, Universitas Padjadjaran, Jatinangor 45363, West Java, Indonesia

**Keywords:** *Lansium domesticum*, Meliaceae, MCF-7, onoceranoid triterpene, tetranortriterpenoid

## Abstract

*Lansium domesticum* Corr. is a member of the Meliaceae family that is widely spread in tropical and subtropical region of Asia and America. Traditionally, the fruit of this plant has been consumed because of its sweet taste. However, the fruit peels and the seeds of this plant have been rarely utilized. The previous chemical investigation of this plant showed the presence of secondary metabolites with many biological activities, including cytotoxic triterpenoid. Triterpenoids is a class of secondary metabolites which contain thirty carbon atoms in the main skeleton. The high modification of this type of compound, including the ring opening, highly oxygenated carbons, and the degradation of its carbon chain to give the nor-triterpenoid structure, is responsible for its cytotoxic activity. In this paper, we isolated and elucidated the chemical structure of two new onoceranoid triterpenes, kokosanolides E (**1**) and F (**2**), from the fruit peels of *L. domesticum* Corr., along with a new tetranortriterpenoid, kokosanolide G (**3**), from the seeds of *L. domesticum* Corr. The structural determination of compounds **1–3** was undertaken through FTIR spectroscopic analysis, 1D and 2D NMR, mass spectrometry, as well as through a comparison of the chemical shifts of the partial structures of compounds **1–3** with the literature data. The cytotoxic properties of compounds **1–3** were tested against MCF-7 breast cancer cells using the MTT assay. Moderate activity was shown by compounds **1** and **3**, with IC_50_ values of 45.90 and 18.41 μg/mL, respectively, while compound **2** showed no activity (IC_50_ 168.20 μg/mL). For the onoceranoid-type triterpene, the high symmetrical structure of compound **1** is presumably the reason for its better cytotoxic activity compared with that of compound **2**. Compound **3** showed moderate activity, mainly because of the presence of the furan ring, which, based on the literature, gives better cytotoxic activity in a tetranortriterpenoid-type structure. The findings of three new triterpenoid compounds from *L. domesticum* indicate the significant value of this plant as a source of new compounds.

## 1. Introduction

*Lansium domesticum* Corr., as one of plant species from the Meliaceae family, is a source of triterpenoid compounds with various biological activities. This plant is widely distributed in Southeast Asia [1,2], Suriname, Puerto Rico, and Australia [3]. The latest taxonomic system of Meliaceae does not assign a specific rank for *L. domesticum* [4]. However, there are three varieties of *L. domesticum* that are widely known in Java, Indonesia, namely, duku, bidjitan or langsat, and kokossan. Duku has small, ellipsoidal, glabrous, pale yellow fruits without latex from trees with glabrous leaves and small flowers. Meanwhile, bidjitan or langsat has larger, ellipsoidal, glabrescent, pale yellow fruits with a little latex from trees with larger flowers and leaves, which are ±pilose abaxially. Kokossan has smaller, globose, orange–yellow fruits with latex and a tough pericarp from trees with the largest flowers and the most pubescent leaves. Hasskarl in Mabberley et al. (1995) [5] proposed the vernacular names as subdivisions of *Lansium domesticum* in Java, and these could be taken as cultivar names. Therefore, *L. domesticum* cv. *kokossan* or *L. domesticum* ‘kokossan’ were used to refer to the varieties of kokossan. 

Almost all parts of *L. domesticum* plants have long been widely used for traditional therapeutic purposes. Boiled water from the bark of *L. domesticum* Corr., which is mixed with the bark of *Pterocarpus indica* Wild, is commonly used as a medicine for dysentry. The green seeds are very bitter, and when crushed with water, they can be used as an anthelmintic and as antipyretics. The stems can be used to treat malaria. The resin is thought to be non-toxic and useful for stopping diarrhea. The leaf extract of *L. domesticum* can be used as eye drops to prevent inflammation. The fruit skin of the plants can be used as a mosquito repellent through the smoke of the burning dry fruit skin. The fruit flesh and tree trunks are used by traditional communities for poisoning arrows [6].

A number of studies have reported various types of triterpenoids isolated from the plant of *L. domesticum* Corr. [7,8,9,10,11,12,13]. These compounds have various biological activities, including antifeedant [14], antimicrobial [15], antimalarial [16], anticancer [17], insecticidal [18], and antimutagenic activities [19,20]. In our continuing effort to find potential compounds from the plant of *L. domesticum* Corr. cv *kokossan*, four onoseranoid triterpenes, a cycloartananoid, and three tetranortriterpenoids have been reported from the barks and peels [14,21], the leaves [22], and the peels and seeds [14,23] of *L. domesticum* Corr. cv *kokossan*, respectively. 

**Figure 1:**
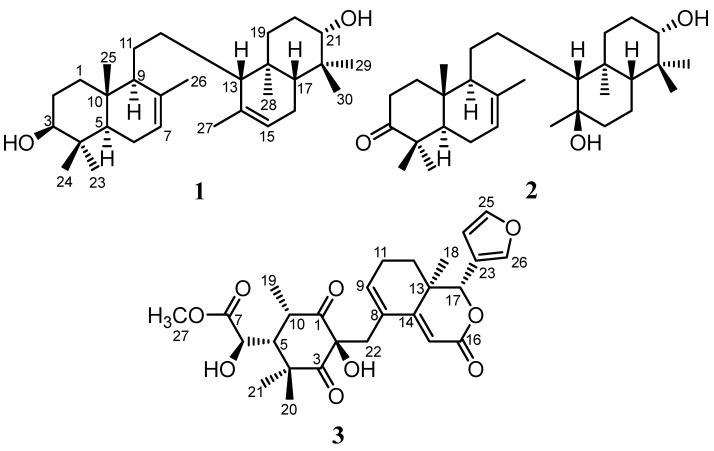
The chemical structures of compounds **1–3**.

In this article, we report the isolation, using various chromatography techniques, and the structure determination, using spectroscopic methods, of two new onoceranoid triterpenes, 3,21-dihydroxy-onocera-7,14-diene (kokosanolide E, **1**) and 14*β*,21*α*-dihydroxy-onocera-7-en-3-one (kokosanolide F, **2**), from the fruit peels, and a new tetranortriterpenoid, kokosanolide G (**3**), from the seeds of *L. domesticum* Corr. cv. *kokossan* (Figure 1). The cytotoxic properties of compounds **1**–**3** were also tested in relation to breast cancer MCF-7 cell lines using an MTT assay. The brief structure activity–relationship of isolated compounds **1**–**3** was also explained. 

## 2. Results and Discussions

After the separation of fruit peels and seeds from the fruit of *L. domesticum*, the drying in room temperature was carried out, followed by grinding, to give 1.7 kg powder of fruit peels and 3.8 kg powder of seeds. Two different extraction techniques were applied for the fruit peels and seeds due to the amount of material used. 

The powder of fruit peels was extracted exhaustively with *n*-hexane, EtOAc, and MeOH. The *n*-hexane extract was chosen for the next separations and purifications due to the presence of the triterpenoid compound, which was identified qualitatively by the Liebermann–Burchard test. After the series of column chromatography (normal and reversed phase), compounds **1** and **2** were obtained.

The powder of seeds was extracted exhaustively with methanol. After the solvent removal under reduced pressure, the methanol extract was dissolved in water and then partitioned by *n*-hexane, EtOAc, and *n*-BuOH. The EtOAc extract was chosen for the next separations and purifications due to the presence of the triterpenoid compound, which was identified qualitatively by the Liebermann–Burchard test. After the series of column chromatography (normal and reversed phase), compound **3** was obtained.

The structure elucidation of the new triterpenoid compounds **1–3** was discussed based on the spectroscopic data and a comparison with the literature, along with its cytotoxic assay against MCF-7 breast cancer cell lines. 

### 2.1. Structure Elucidation of the Isolated Compounds

**Figure 2:**
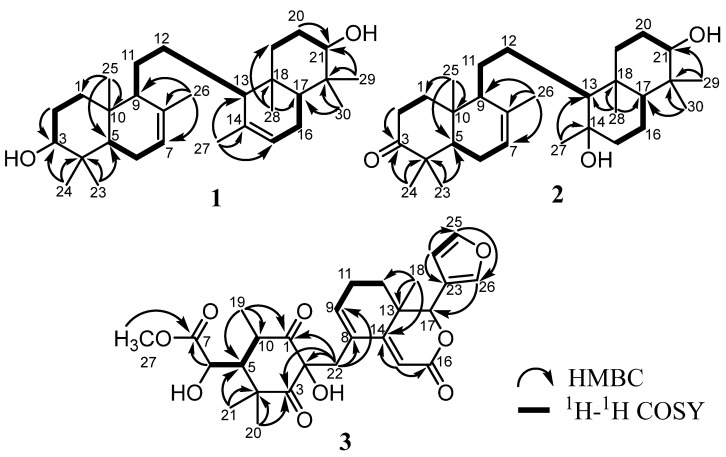
Selected HMBC and ^1^H–^1^H COSY correlations for **1–3**.

Compound **1** was discovered as an amorphous and colorless powder. According to the NMR data and HRTOFMS data results that indicated a molecular ion peak at *m/z* 443.3881 [M+H]^+^ (calculated for C_30_H_51_O_2_ at *m/z* 443.3889), the molecular formula of **1** was determined to be C_30_H_50_O_2_, with six degrees of unsaturation. Fifteen carbon signals, including four methyls, four methylenes, four methines (involving one olefinic and one oxygenated carbon), and three quaternary carbons (including one olefinic carbon), were identified through ^13^C NMR (DEPT) data (Table 1) and HSQC spectra. The IR spectrum suggested the existence of hydroxyl (3448 cm^−1^), *gem*-dimethyl (1385 and 1458 cm^−1^), and ether (1022 cm^−1^) functionalities. The above data revealed that **1** is a symmetric onoceranoid-type triterpenoid with a total of 30 carbon signals [7]. The two olefinic groups accounted for two degrees of unsaturation, leaving four degrees of unsaturation for the four-rings core of **1**. In the ^1^H NMR spectrum, eight tertiary methyls at δ_H_ 0.74 (6H, s, Me-25 and Me-28), 0.85 (6H, s, Me-23 and Me-29), 0.98 (6H, s, Me-24 and Me-30), and 1.70 (6H, s, Me-26 and Me-27), two olefinic protons at δ_H_ 5.39 (2H, brs, H-7 and H-15), and two oxymethines at δ_H_ 3.26 (2H, dd, *J* = 11.0 and 4.0 Hz, H-3 and H-21) were obviously observed. Detailed analysis of the 2D NMR spectra (^1^H-^1^H COSY and HMBC) (Figure 2) defined the structure of **1**. The correlations of ^1^H-^1^H COSY gave cross-peaks of H-2/H-3, H-5/H-6, H-6/H-7, and H-9/H-11, showing the presence of an onoceranoid-type triterpene framework [7]. The two hydroxyl methines positioned at C-3 and C-21 were deduced by the HMBC correlations between Me-23/Me-24 to C-3 and Me-29/Me-30 to C-21. Furthermore, the correlations of Me-26 to C-7, C-8, and Me-27 to C-14, C-15 verified the formation of two double-bond pairs at C-7/C-8 and C-14/C-15. A careful analysis of the 1D and 2D NMR showed that **1** was similar to 3*β*-hydroxyonocera-8(26),14-dien-21-one [10], with the main differences being the double-bond movement at C-7/C-8 and C-14/C-15 in the B ring and the ketonic replacement at C-21 by a hydroxyl moiety. In addition, the hydroxyl group on C-3 (C-21) was determined to be an *α*-orientation by the *J* coupling value compared to the 3β-hydroxyl in its analog [10], referencing the reflection form of the stereocenter’s orientation in the symmetrical structure of the onoceranoid-type. Thus, compound **1** was elucidated as a new onoceranoid triterpene derivative, 3,21-dihydroxy-onocera-7,14-diene, and trivially named as kokosanolide E (**1**).

Compound **2** was discovered as an amorphous and colorless powder. Referencing the NMR data and the HRTOFMS data results that showed a molecular ion peak at *m*/*z* 459.3844 [M+H]^+^ (calculated for C_30_H_51_O_3_ at *m*/*z* 459.3838), the molecular formula of **2** was established as C_30_H_50_O_3_, with six degrees of unsaturation. The IR spectrum exhibited hydroxyl (3443 cm^–1^), C-H stretching of aliphatic carbon (2939 cm^–1^), ketone (1702 cm^–1^), *gem*-dimethyl (1460 and 1386 cm^–1^), and ether (1040 cm^–1^) groups. The ^13^C NMR analysis with the aid of the DEPT and HSQC spectra of **2** (Table 1) showed the presence of a total of thirty carbon signals, which were assigned as eight methyls, nine methylenes, six methines (involving one olefinic and one oxygenated carbon), and seven quaternary carbons (including one olefinic and two oxygenated carbons), together with the aid of DEPT and HSQC spectra. The above data suggested that **2** is an onoceranoid-type triterpenoid [7]. The presence of one double-bond pair and one carbonyl ketone accounted for two degrees of unsaturation, leaving four degrees of unsaturation for the four-rings core of **2**. In the ^1^H NMR spectrum, eight tertiary methyls at δ_H_ (ppm) 1.09 (3H, Me-23), 1.04 (3H, Me-24), 0.96 (3H, Me-25), 1.76 (3H, Me-26), 1.15 (3H, Me-27), 0.77 (3H, Me-28), 0.99 (3H, Me-29), and 0.75 (3H, Me-30), one olefinic methine at δ_H_ 5.40 ppm (2H, brs, H-7), and one oxygenated methine at δ_H_ 3.23 ppm (2H, dd, *J* = 11.5 and 4.0 Hz, H-21) were clearly identified. Detailed analysis of the 2D NMR spectra (^1^H-^1^H COSY and HMBC) (Figure 2) defined the structure of **2.** The ^1^H-^1^H COSY showed correlations of H-1/H-2, H-6/H-7, H-15/H-16/H-17, and H-19/H-20/H-21, confirming an onoceranoid-type triterpene framework [7]. The presence of a ketonic group at C-3 and a hydroxyl methine group at C-21 was verified by the correlations between Me-23/Me-24 to C-3 and Me-29/Me-30 to C-21. Furthermore, the correlations of Me-27 to C-14 and Me-26 to C-7, C-8 revealed the attachment of a hydroxyl in the quaternary carbon at C-14 and the double-bond form at C-7/C-8. A careful analysis of the 1D and 2D NMR indicated that compound **2** was closely related to kokosanolide B [14], with the main difference being the hydroxyl moiety at C-21. The relative configuration of each stereocenter carbon in **2** was determined by an NOESY experiment. The NOESY spectrum showed correlations between H-21/H-17/H-3 and H-28/H-27. According to the biosynthesis of the onoceranoid-type triterpene, the orientation of H-17 is -oriented, which indicates that the H-21 proton has a orientation. Based on this result, it can be concluded that the hydroxyl group at C-21 is α-oriented, while the hydroxyl group at C-14 is -oriented. Thus, compound **2** was elucidated as a new onoceranoid-type triterpene, 14,21α-dihydroxy-onocera-7-en-3-one, and trivially named as kokosanolide F (**2**).

Compound **3** was originally discovered as a colorless oil. According to the NMR data (Table 1), together with its HR-TOFMS analysis, compound **3** showed an [M+H]^+^ ion peak at *m/z* 501.2125 (calcd. 501.2131 for C_27_H_33_O_9_), which was consistent with the formula C_27_H_32_O_9_, requiring 12 degrees of unsaturation. The UV spectrum suggested the presence an α, -unsaturated ketone by an absorption maximum at 282 nm. The IR spectrum showed the existence of a hydroxyl at 3453 cm^−1^, a carbonyl ketone at 1707 cm^−1^, an ester at 1670 cm^−1^, a *gem*-dimethyl at 1367 and 1379 cm^−1^, and an ether at 1278 cm^−1^. In the ^1^H-NMR spectrum, three tertiary methyls at δ_H_ 0.91, 0.95, and 1.07 and one secondary methyl at δ_H_ 1.27 (d, *J* = 6.4 Hz), as well as the downfield tertiary methyl group, resonating at δ_H_ 3.80, indicated the presence of a methoxy group that was identified. The signals corresponding to a tetranortriterpenoid bearing an α-substituted furan ring at δ_H_ 6.41, 7.40, and 7.44 were obviously observed. Moreover, the signal for an olefinic of an α,β-unsaturated ketone at δ_H_ 5.97 (1H, m), another olefinic signal at δ_H_ 6.39 (1H, t, *J* = 1.0 Hz), and two protons corresponding to the oxymethine group at δ_H_ 4.32 (1H, d, *J* = 1.0 Hz) and 5.02 (1H, s) were also found. The ^13^C-NMR (DEPT) data revealed a total of twenty-seven carbon signals, including characteristics of a furan moiety (δ_C_ 143.1 (d), 141.1 (d), 120.0 (s), and 110.2 (d)), two ketonic groups (δ_C_ 211.5 and 208.1), two ester groups (δ_C_ 174.1 and 166.3), two oxymethine carbons (δ_C_ 73.8 and 80.8), one quaternary oxygenated carbon (δ_C_ 80.4), a double-bond at an unsaturated ketone (δ_C_ 159.7 and 110.4), and an additional double-bond (δ_C_ 141.5 and 126.7). According to the 1D NMR data, the functionality of **3** accounted for eight degrees of unsaturation, leaving four degrees of unsaturation suitable for a tetracyclic tetranortriterpenoid core with a furan ring. The main skeleton of the tetranortriterpenoid compound was further proven by the ^1^H-^1^H COSY cross-peaks of H-24/H-25, H-9/H-11/H-12, and H-6/H-5/H-10. The study of the kokosanolide-type tetranortriterpenoid skeleton in the genus of *Lansium* revealed that the NMR data of **3** were related to the kokosanolide A isolated from the same species [14]. The difference featuring the ether ring opening (C-1/C-9) in kokosanolide A, which resulted in the formation of a double-bond at C-8/C-9 of **3**, was indicated by the HMBC correlation of H-22 to C-8 and C-9, as well as the ^1^H-^1^H COSY of H-9/H-11. This one ring opening also led to the formation of an additional carbonyl group at C-1, which was observed through a correlation of H-22 and H-19 to C-1. Other structural characteristics of **3** are similar to the characteristics of kokosanolide A, including the formation of a lactone ring at C-16/C-17, which was shown by the correlation of H-15 to C-16, and the presence of a methyl ester at C-27, which arose from the correlation of H-27 to C-7. Finally, the furan moiety at C-17 was evidenced by the correlation of H-26 to C-17. The relative stereochemistry of **3** was mainly determined by the similarity of the ^1^H and ^13^C NMR chemical shifts, the *J* coupling constants, as well as the NOESY spectrum of kokosanolide A. From the NOESY spectrum, a cross-peak arising at H-6/H-5/H-10 indicated that those three protons were in the same face. Therefore, the structure of **3** was elucidated as the new tetranortriterpenoid and trivially named as kokosanolide G (**3**).

### 2.2. Cytotoxic Activity of Isolated Compounds

Compounds **1–3** were evaluated for their cytotoxic activity against MCF-7 breast cancer cell lines and compared to doxorubicin (0.17 μg/mL) as a positive control. The IC_50_ values of compounds **1–3** are 45.90, 168.20, and 18.41 μg/mL (Tables S4–S6), respectively. Compounds **1** and **3** exhibited moderate activity against MCF-7, while compound **2** showed no activity. The significant difference in cytotoxic activity between the onoceranoid triterpenes **1** and **2** was most probably due to the symmetrical structure of **1** compared to the structure of **2**. Additionally, the presence of a carbonyl group at **2** was expected to decrease the cytotoxic activity. Compound **3** showed the highest cytotoxic activity among all isolated compounds, probably due to the presence of a furan ring and a highly oxygenated structure.

## 3. Materials and Methods

### 3.1. General Experimental Procedures

High-resolution electrospray ionization (HRESIMS) was acquired on a waters Xevo QTOFMS (Waters, Milford, MA, USA). IR spectra were performed on a One Perkin Elmer infrared-100. NMR spectra were obtained on a JEOL ECZ-500 spectrometer at 500 MHz for ^1^H NMR and at 125 MHz for ^13^C NMR, with tetramethylsilane (TMS) as the internal reference. For the column chromatography, silica gel G60 (Merck, Darmstadt, Germany) and C18 silica gel (Merck, Darmstadt, Germany) were used. TLC was performed on precoated GF_254_ (Merck, 0.25 mm) silica gel plates, and TLC spots were detected using 10% sulfuric acid in ethanol and then heated.

### 3.2. Plant Material

The seeds and fruit peels of *L. domesticum* were collected in April 2018 from Cililin, West Java, Indonesia (6°57′2″ S, 107°27′25″ E, 667 msl). A plant specimen (10188), identified by the staff of the Laboratory of Plant Taxonomy, was deposited in the Department of Biology, Universitas Padjadjaran, Indonesia. 

### 3.3. Extraction and Isolation

The dried fruit peels of *L. domesticum* (1.7 kg) were exhaustively macerated with *n*-hexane (6 × 2 L), EtOAc (5 × 2 L), and MeOH (5 × 2 L) at room temperature. The crudes of *n*-hexane extract (86 g), EtOAc extract (110 g), and MeOH extract (75 g) were obtained through solvents evaporation. Vacuum liquid chromatography (VLC) was then used to fractionate the *n*-hexane extract (86 g) on silica gel using a 10% gradient of *n*-hexane-EtOAc-MeOH to afford eight fractions (A–H). Fraction G (2.3 g) was fractionated by VLC on silica gel using a 1% gradient of CHCl_3_-MeOH to give five fractions (G1–G5). Fraction G4 (963.3 mg) was separated over a silica gel column using a 5% gradient of *n*-hexane-EtOAc to yield thirteen subfractions (G4.1–G4.13). Subfractions G4.4 and G4.5 were combined (130.1 mg) and separated on a silica gel column using CH_2_Cl_2_-EtOAc (8:2) to give four combined subfractions (G4.4.1–G4.4.4). Subfractions G4.4.2–G4.4.4 were amalgamated and further purified by a column of RP-18 silica gel using MeOH-H_2_O with 2% gradient to yield **1** (3.1 mg). Fraction F (1.5 g) was fractionated by a column of RP-18 silica gel using 2% gradient of MeOH-H_2_O to give three fractions (F1–F3). Fraction F1 (636 mg) was separated over a silica gel column eluted with 1% gradient of CH_2_Cl_2_-EtOAc to give four subfractions (F1.1–F1.4). Fraction F1.4 (107 mg) was then subjected on a silica gel column eluted with 2% gradient of *n*-hexane-EtOAc to yield three subfractions (F1.4.1–F1.4.3). Fraction F1.4.2 (63 mg) was purified by a silica gel column with 2% gradient of *n*-hexane-EtOAc to yield **2** (28 mg). We used several known onoceranoid triterpenes as the standard on TLC plates in almost every step of the purification process. This step was carried out to avoid isolating known compounds. Fraction F and G were chosen based on the TLC profiles, showing new TLC spots and indicating the presence of new compounds.

The dried seeds of this species (3.8 kg) were extracted exhaustively with MeOH (5 × 5 L) at room temperature. The evaporation of the organic layer gave a concentrated MeOH extract (143.5 g). The crude MeOH extract was partitioned between H_2_O and *n*-hexane, EtOAc, and *n*-BuOH, successively. The EtOAc soluble fraction (10 g) was fractionated by VLC on silica gel using a stepwise gradient of *n*- hexane-EtOAc 5% to yield eight fractions (I-P). Fraction L (1.38 g) was chromatographed by a silica gel column (*n*-hexane-CH_2_Cl_2_-EtOAc, 4:3:3) to give six fractions (L1–L6). The separation of subfraction L4 (324 mg) by a silica gel column with a stepwise gradient elution of *n*-hexane-EtOAc 5% gave three subfractions (L4.1–L4.3). Subfraction L4.3 (106 mg) was then purified by a silica gel column (*n*-hexane-CH_2_Cl_2_-EtOAc, 4.5:4:1.5) to yield **3** (9.8 mg). Fraction L was chosen based on TLC profiles because there was a new spot, which indicated the presence of a new compound.

#### 3.3.1. Kokosanolide E (**1**)

Colorless amorphous powder: [λ]^25^_D_ −10 (c 0.5, MeOH); IR (KBr) υ_max_ 3448, 2963, 1385, 1458, 1022 cm^−1^; HR-TOFMS *m/z* 443.3881 [M+H]^+^ (calcd. for C_30_H_51_O_2_, *m/z* 443.3889); ^1^H-NMR (CDCl_3_, 500 MHz); and ^13^C-NMR (CDCl_3_, 125 MHz) data are shown in Table 1.

#### 3.3.2. Kokosanolide F (**2**)

Colorless amorphous powder: [λ]^25^_D_ −15 (c 0.5, MeOH); IR (KBr) υ_max_ 3443, 2939, 1702, 1460, 1386, 1040 cm^−1^; HR-TOFMS *m/z* 459.3844 [M+H]^+^ (calcd. for C_30_H_51_O_3_, *m/z* 459.3838); ^1^H-NMR (CDCl_3_, 500 MHz); and ^13^C-NMR (CDCl_3_, 125 MHz) data are shown in Table 1.

#### 3.3.3. Kokosanolide G (**3**)

Colorless oil: [λ]^25^_D_ +65 (c 0.5, CHCl_3_); UV (MeOH) λ_max_ (log e): 224 (4.09), 286 (4.12) nm; IR (KBr) υ_max_ 3453, 2975, 1707, 1670, 1367, 1379, 1278 cm^−1^; HR-TOFMS *m/z* 501.2125 [M+H]^+^ (calcd. for C_27_H_33_O_9_, *m/z* 501.2131); ^1^H-NMR (CDCl_3_, 500 MHz); and ^13^C-NMR (CDCl_3_, 125 MHz) data are shown in Table 1.

### 3.4. Cytotoxic Bioassay

All isolated compounds were tested for their cytotoxicity against human breast cancer cells (MCF-7) using the MTT (methyl thiazoldiphenyl-tetrazoliumbromide) method. The cells were cultured in Roswell Park Memorial Institute (RPMI) Medium (DMEM), 10% (*v*/*v*) Fetal Bovine Serum (FBS), and an antibiotic of 1% (*v*/*v*) (100U) penicillin-streptomycin mixture solution. The incubation was carried out at 37 °C for 24 h. The media were replaced by a mixture of fresh media with the addition of the isolated compounds at various concentrations (7.81; 15.63; 31.25; 62.50; 125.00; 250.00; 500.00; 1000.00 µg/mL). After 24 h, the mixture of 200 μL of DMSO and the formed formazan crystal was used to replace the media from each well. The absorbance was measured at 450 nm, and the IC_50_ can be calculated through linier regression with Microsoft Excel software.

## 4. Conclusions

Three new triterpenoids, kokosanolides E-G (**1–3**), were isolated from *L. domesticum* Corr. Compounds **1–2** were isolated from the fruit peels part, whereas compound **3** was isolated from the seeds part. Extensive spectroscopic methods were used for the determination of the chemical structure of **1–3**. Compounds **1** and **2** belong to an onoceranoid triterpene and compound **3** has a tetranortriterpenoid structure. All of the isolated compounds were tested for cytotoxic activity against the MCF-7 breast cancer cell line using MTT methods, which showed that compounds **1** and **3** have moderate activity, whereas compound **2** has no activity against MCF-7 cell lines. The highly symmetrical structure of **1** and the highly oxygenated nature and the presence of a furan ring in **3** are suspected to play important roles in cytotoxic activity.

## Figures and Tables

**Table 1:** NMR Data (500 MHz for ^1^H and 125 MHz for ^13^C, in CDCl_3_) for **1–3**.

No.	1		2		3	
	^13^C NMR δc	^1^H NMR δ_H_ (Integral, Mult, *J* = Hz)	^13^C NMR δc	^1^H NMR δ_H_ (Integral, Mult, *J* = Hz)	^13^C NMR δc	^1^H NMR δ_H_ (Integral, Mult, *J* = Hz)
1	30.1	1.25, 1.45 (each 1H, m)	38.3	1.44, 2.10 (each 1H, m)	211.5	-
2	27.6	1.62, 1.66 (each 1H, m)	34.9	2.26, 2.72 (each 1H, m)	80.4	-
3	79.3	3.26 (1H, dd, 11.0, 4.0)	217.4	-	208.1	-
4	38.9	-	47.6	-	47.3	-
5	49.8	1.20 (1H, m)	51.6	1.57 (1H, m)	53.0	2.18 (1H, dd, 1.5, 1.0)
6	23.7	1.97 (2H, m)	28.8	1.40, 1.84 (each 1H, m)	73.8	4.32 (1H, d, 1.0)
7	122.1	5.39 (1H, brs)	121.6	5.40 (1H, brs)	174.1	-
8	135.4	-	135.9	-	126.7	-
9	56.3	1.59 (1H, m)	55.4	1.59 (1H, m)	141.5	6.39 (1H, t, 1.0)
10	36.7	-	38.7	-	43.2	3.06 (1H, m)
11	37.6	1.17, 1.88 (each 1H, m)	20.4	1.32, 1.66 (each 1H, m)	22.5	2.30 (2H, m)
12	37.6	1.17, 1.88 (each 1H, m)	24.2	1.22, 1.88 (each 1H, m)	29.7	1.39 (2H, m)
13	56.3	1.59 (1H, m)	62.6	0.99 (1H, m)	37.7	-
14	135.4	-	74.3	-	159.7	-
15	122.1	5.39 (1H, brs)	44.8	1.36, 1.86 (each 1H, m)	110.4	5.97 (1H, m)
16	23.7	1.97 (2H, m)	31.5	1.36, 1.40 (each 1H, m)	166.3	-
17	49.8	1.20 (1H, m)	55.2	0.88 (1H, m)	80.8	5.02 (1H, s)
18	36.7	-	36.6	-	15.9	0.95 (3H, s)
19	30.1	1.25, 1.45 (each 1H, m)	38.0	1.10, 1.65 (each 1H, m)	16.0	1.27 (3H, d, 6.4)
20	27.6	1.62, 1.66 (each 1H, m)	27.3	1.59, 1.68 (each 1H, m)	26.5	0.91 (3H, s)
21	79.3	3.26 (1H, dd, 11.0, 4.0)	78.9	3.23 (1H, dd, 11.5; 4.0)	20.2	1.07 (3H, s)
22	38.9	-	39.0	-	36.1	2.86, 3.19 (each 1H, s)
23	15.3	0.85 (3H, s)	22.3	1.09 (3H, s)	120.0	-
24	28.1	0.98 (3H, s)	25.1	1.04 (3H, s)	110.2	6.41 (1H, d, 2.0)
25	13.8	0.74 (3H, s)	13.4	0.96 (3H, s)	143.1	7.40 (1H, d, 2.0)
26	22.6	1.70 (3H, s)	22.3	1.76 (3H, s)	141.1	7.44 (1H, s)
27	22.6	1.70 (3H, s)	24.3	1.15 (3H, s)	53.0	3.80 (3H, s)
28	13.8	0.74 (3H, s)	15.8	0.77 (3H, s)		
29	15.3	0.85 (3H, s)	28.3	0.99 (3H, s)		
30	28.1	0.98 (3H, s)	15.5	0.75 (3H, s)		

## Data Availability

All the data in this research are presented in the manuscript and the Supplementary Material.

## Supplementary Materials

The following supporting information can be downloaded at: https://www.mdpi.com/article/10.3390/molecules28052144/s1, Figure S1: IR spectrum of 1; Figure S2: HR-ESI-MS spectrum of 1; Figure S3: ^1^H-NMR (500 MHz, CDCl_3_) spectrum of 1; Figure S4: ^13^C-NMR (125 MHz, CDCl_3_) spectrum of 1; Figure S5: DEPT135 (125 MHz, CDCl_3_) spectrum of 1; Figure S6: HMQC spectrum of 1; Figure S7: HMQC spectrum of 1 (From *δ*_C_10 ppm to *δ*_C_ 60 ppm); Figure S8: HMBC spectrum of 1; Figure S9: HMBC spectrum of 1 (From *δ*_C_ 10 ppm to *δ*_C_ 60 ppm); Figure S10: HMBC spectrum of 1 (From *δ*_C_ 10 ppm to *δ*_C_ 140 ppm); Figure S11: ^1^H-^1^H COSY spectrum of 1; Figure S12: IR spectrum of 2; Figure S13: HR-ESI-MS spectrum of 2; Figure S14: ^1^H-NMR (500 MHz, CDCl_3_) spectrum of 2; Figure S15: ^13^C-NMR (125 MHz, CDCl_3_) spectrum of 2; Figure S16: DEPT135 (125 MHz, CDCl_3_) spectrum of 2; Figure S17: HMQC spectrum of 2; Figure S18: HMQC spectrum of 2 (From *δ*_C_ 30 ppm to 70 ppm); Figure S19: HMQC spectrum of 2 (From *δ*_C_ 13 ppm to 32 ppm); Figure S20: HMBC spectrum of 2; Figure S21: HMBC spectrum of 2 (expansion); Figure S22: HMBC spectrum of 2 (From *δ*_C_ 40 ppm to 80 ppm); Figure S23: HMBC spectrum of 2 (From *δ*_C_ 10 ppm to 80 ppm); Figure S24: ^1^H-^1^H COSY spectrum of 2; Figure S25: NOESY spectrum of 2; Figure S26: UV spectrum of 3; Figure S27: IR spectrum of 3; Figure S28: HR-ESI-MS spectrum of 3; Figure S29: ^1^H-NMR (500 MHz, CDCl_3_) spectrum of 3; Figure S30: ^13^C-NMR (125 MHz, CDCl_3_) spectrum of 3; Figure S31: DEPT135 (125 MHz, CDCl_3_) spectrum of 3; Figure S32: HMQC spectrum of 3; Figure S33: HMQC spectrum of 3 (From *δ*_C_ 15 ppm to 31 ppm); Figure S34: HMBC spectrum of 3; Figure S35: HMBC spectrum of 3 (From *δ*_C_ 10 ppm to 60 ppm); Figure S36: ^1^H-^1^H COSY spectrum of 3; Figure S37: NOESY spectrum of 3; Figure S38: Scifinder reports of compounds 1; Figure S39: Scifinder reports with a similarity score of 98 to compound 1; Figure S40: Scifinder reports with a similarity score of 95 to compound 1; Figure S41: Scifinder reports of compound 2; Figure S42: Scifinder reports with a similarity score of 99 to compound 2; Figure S43: Scifinder reports with a similarity score of 95 to compound 2; Figure S44: Scifinder reports of compound 3; Figure S45: Scifinder reports with a similarity score of 95 to compound 3; Table S1: NMR data comparison of compound 1 with 3-hydroxy-8,14-secogammacera-7,14-dien-21-one [20]; Table S2: NMR data comparison of compound 2 with kokosanolide B [13]; Table S3: NMR data comparison of compound 3 with kokosanolide A [13]; Table S4: Results of cytotoxic activity of 1 against MCF-7 cell line; Table S5: Results of cytotoxic activity of 2 against MCF-7 cell line; Table S6: Results of cytotoxic activity of 3 against MCF-7 cell line.

## Sample Availability

All samples of the compounds are not available from the authors.
