# Supplementary material for: New Triterpenoids from Lansium domesticum Corr. cv kokossan and Their Cytotoxic Activity
Source: Molecules. 2023 Feb 24;28(5):2144. doi: 10.3390/molecules28052144 (PMC10004713; doi:10.3390/molecules28052144)
Supplement: Supplementary file 1 [file molecules-28-02144-s001.zip › molecules-2200476-supplementary.pdf]

Supporting Information

New triterpenoids from *Lansium domesticum* Corr. cv Kokossan and their cytotoxic activity

Tri Mayanti [1], Zulfikar [1], Sarah Fawziah [1], Al Arofatus Naini [1], Darwati [1], Kindi Farabi [1], Nurlelasari [1], Muhammad Yusuf [1], and Unang Supratman [1,2*]

[1]*Department of Chemistry, Faculty of Mathematics and Natural Sciences, Universitas Padjadjaran, Jatinangor 45363, West Java, Indonesia*
[2]*Central Laboratory, Universitas Padjadjaran, Jatinangor 45363, West Java, Indonesia*

Table of Contents	Page

**Figure S1:** IR spectrum of **1**	3
**Figure S2:** HR-ESI-MS spectrum of **1**	4
**Figure S3:** ^{1}H-NMR (500 MHz, $CDCl_3$) spectrum of **1**	5
**Figure S4:** ^{13}C-NMR (125 MHz, $CDCl_3$) spectrum of **1**	6
**Figure S5:** DEPT135 (125 MHz, $CDCl_3$) spectrum of **1**	7
**Figure S6:** HMQC spectrum of **1**	8
**Figure S7:** HMQC spectrum of **1** (From δ_C10 ppm to δ_C 60 ppm)	9
**Figure S8:** HMBC spectrum of **1**	10
**Figure S9:** HMBC spectrum of **1** (From δ_C 10 ppm to δ_C 60 ppm)	11
**Figure S10:** HMBC spectrum of **1** (From δ_C 10 ppm to δ_C 140 ppm)	12
**Figure S11:** ^{1}H-^{1}H COSY spectrum of **1**	13
**Figure S12:** IR spectrum of **2**	14
**Figure S13:** HR-ESI-MS spectrum of **2**	15
**Figure S14:** ^{1}H-NMR (500 MHz, $CDCl_3$) spectrum of **2**	16
**Figure S15:** ^{13}C-NMR (125 MHz, $CDCl_3$) spectrum of **2**	17
**Figure S16:** DEPT135 (125 MHz, $CDCl_3$) spectrum of **2**	18
**Figure S17:** HMQC spectrum of **2**	19
**Figure S18:** HMQC spectrum of **2** (From δ_C 30 ppm to 70 ppm)	20
**Figure S19:** HMQC spectrum of **2** (From δ_C 13 ppm to 32 ppm)	21
**Figure S20:** HMBC spectrum of **2**	22
**Figure S21:** HMBC spectrum of **2** (expansion)	23
**Figure S22:** HMBC spectrum of **2** (From δ_C 40 ppm to 80 ppm)	24
**Figure S23:** HMBC spectrum of **2** (From δ_C 10 ppm to 80 ppm)	25
**Figure S24:** ^{1}H-^{1}H COSY spectrum of **2**	26
**Figure S25:** NOESY spectrum of **2**	27
**Figure S26:** UV spectrum of **3**	28
**Figure S27:** IR spectrum of **3**	29
**Figure S28:** HR-ESI-MS spectrum of **3**	30
**Figure S29:** ^{1}H-NMR (500 MHz, $CDCl_3$) spectrum of **3**	31
**Figure S30:** ^{13}C-NMR (125 MHz, $CDCl_3$) spectrum of **3**	32
**Figure S31:** DEPT135 (125 MHz, $CDCl_3$) spectrum of **3**	33
**Figure S32:** HMQC spectrum of **3**	34
**Figure S33:** HMQC spectrum of **3** (From δ_C 15 ppm to 31 ppm)	35
**Figure S34:** HMBC spectrum of **3**	36
**Figure S35:** HMBC spectrum of **3** (From δ_C 10 ppm to 60 ppm)	37
**Figure S36:** ^{1}H-^{1}H COSY spectrum of **3**	38
**Figure S37:** NOESY spectrum of **3**	39
**Figure S38:** Scifinder reports of compounds **1.**	40
**Figure S39**: Scifinder reports with similarity score 98 to compounds **1**.	41
**Figure S40**: Scifinder reports with similarity score 95 to compounds **1**	42
**Figure S41**: Scifinder reports of compounds **2**	43
**Figure S42**: Scifinder reports with similarity score 99 to compounds **2**	44
**Figure S43**: Scifinder reports with similarity score 95 to compounds **2**	45
**Figure S44:** Scifinder reports of compounds **3.**	46
**Figure S45**: Scifinder reports with similarity score 95 to compounds **3**	47
**Table S1:** NMR data comparison of compound **1** with 3-hydroxy-8,14-secogammacera-7,14-dien-21-one [20].	48
**Table S2:** NMR data comparison of compound **2** with kokosanolide B [13].	49
**Table S3:** NMR data comparison of compound **3** with kokosanolide A [13].	50
**Table S4**: Results of cytotoxic activity of **1** against MCF-7 cell line.	51
**Table S5**: Results of cytotoxic activity of **2** against MCF-7 cell line.	51
**Table S6**: Results of cytotoxic activity of **3** against MCF-7 cell line.	52
**References**	52

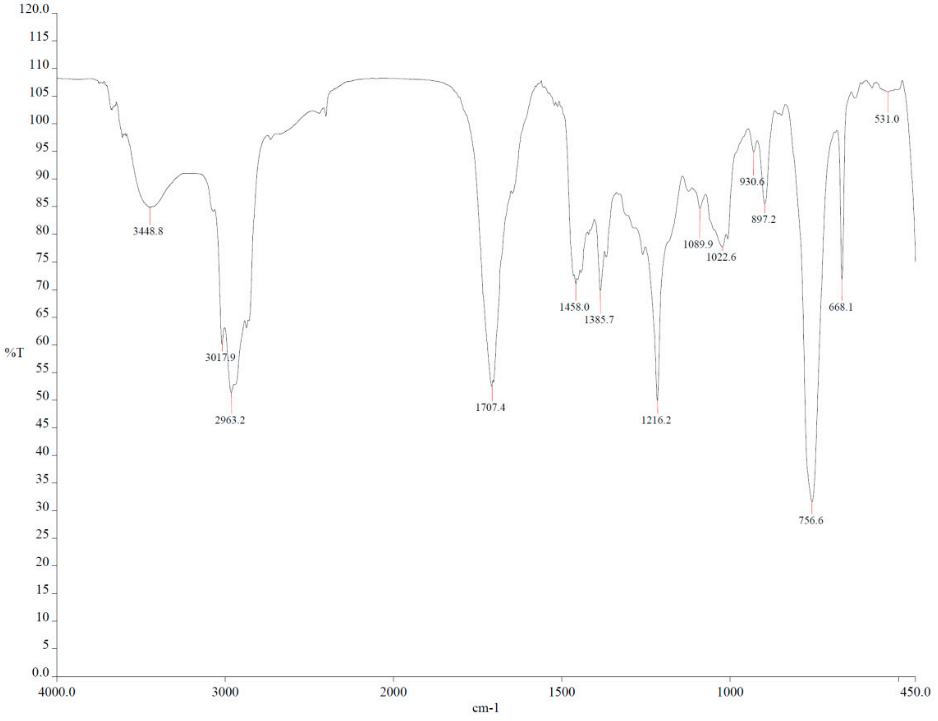

Figure S1 : IR spectrum of **1**

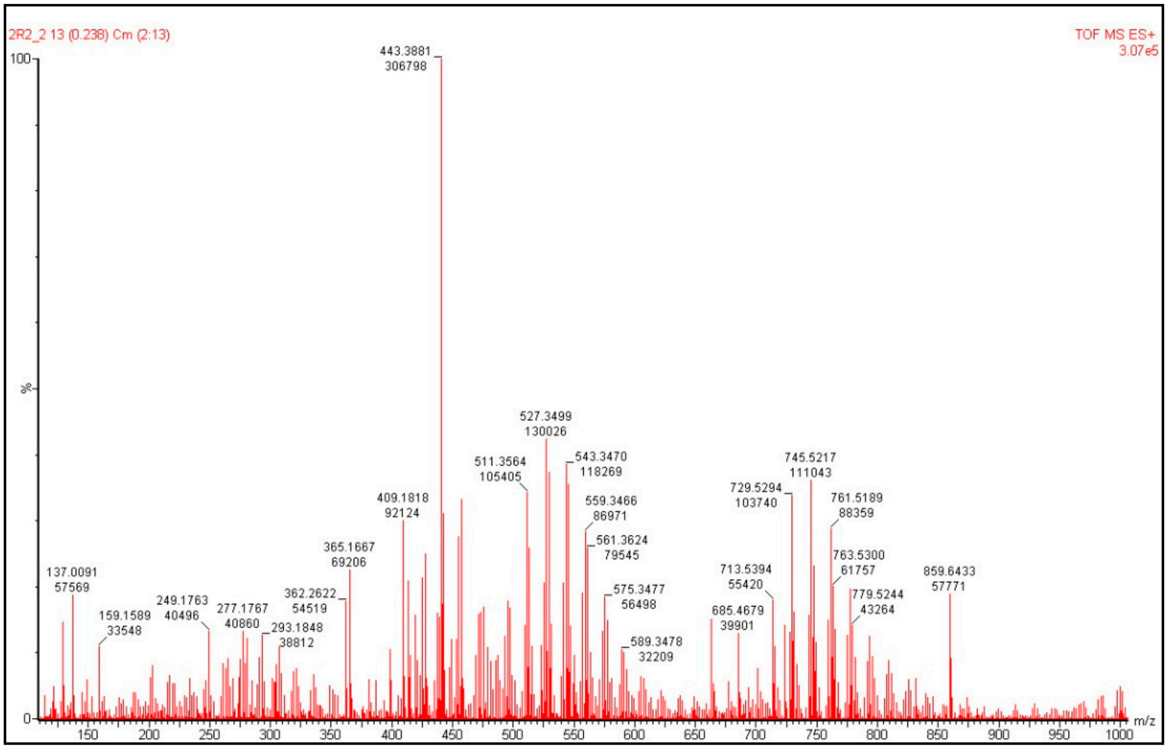

Figure S2: HR-ESI-MS spectrum of **1**.

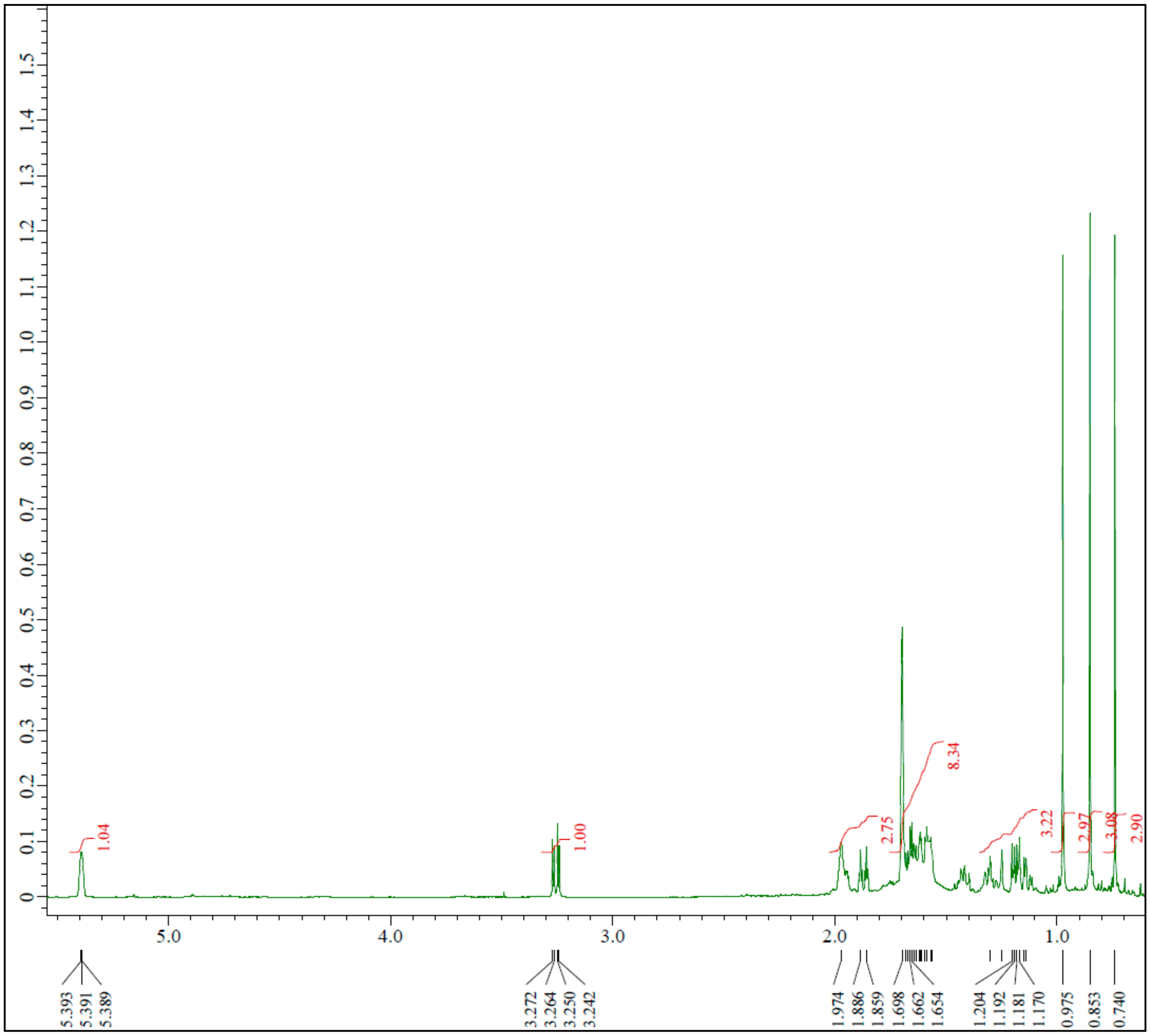

Figure S3: ^{1}H-NMR (500 MHz, $CDCl_3$) spectrum of **1**.

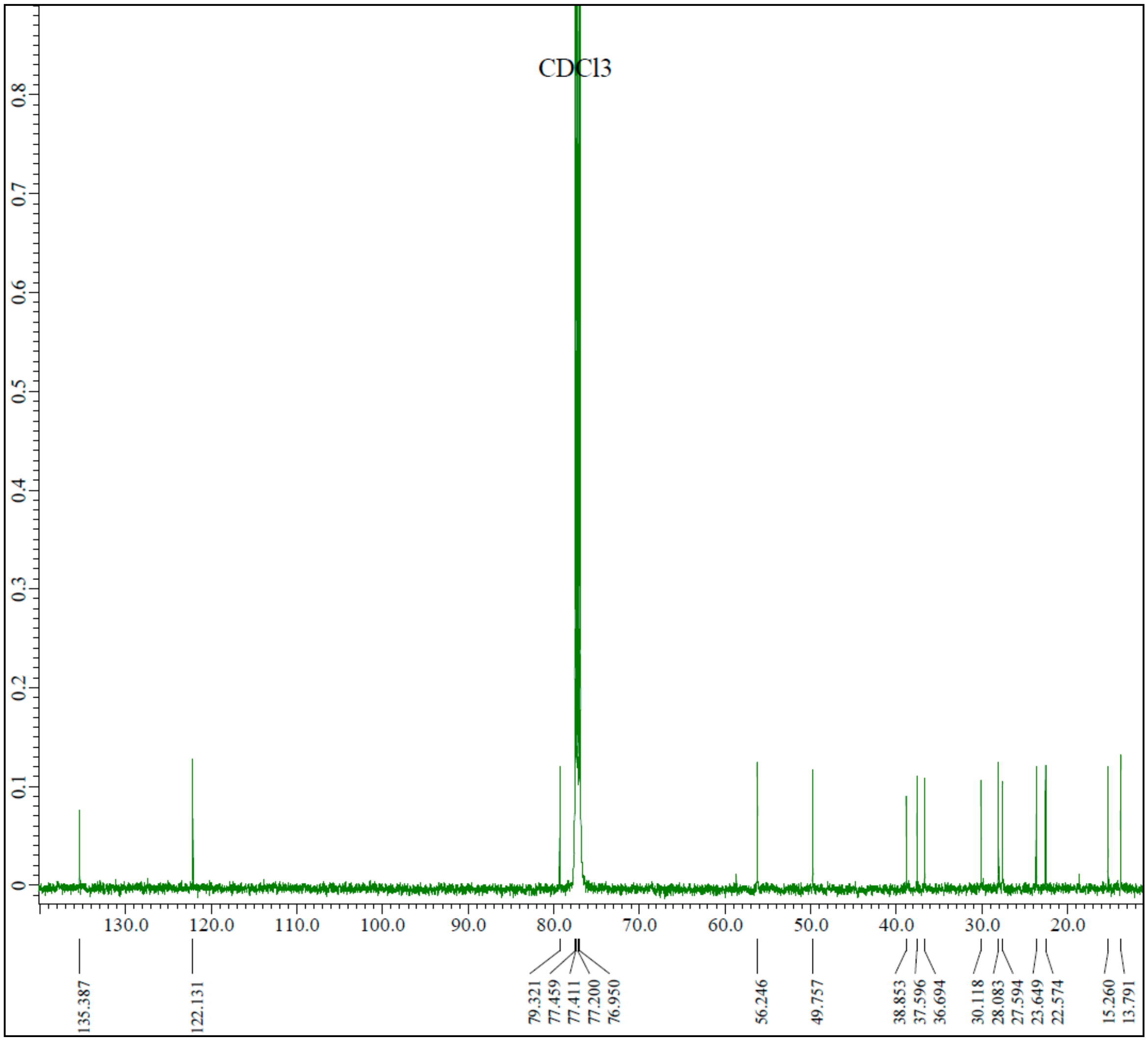

Figure S4: ^{13}C-NMR (125 MHz, $CDCl_3$) spectrum of **1**.

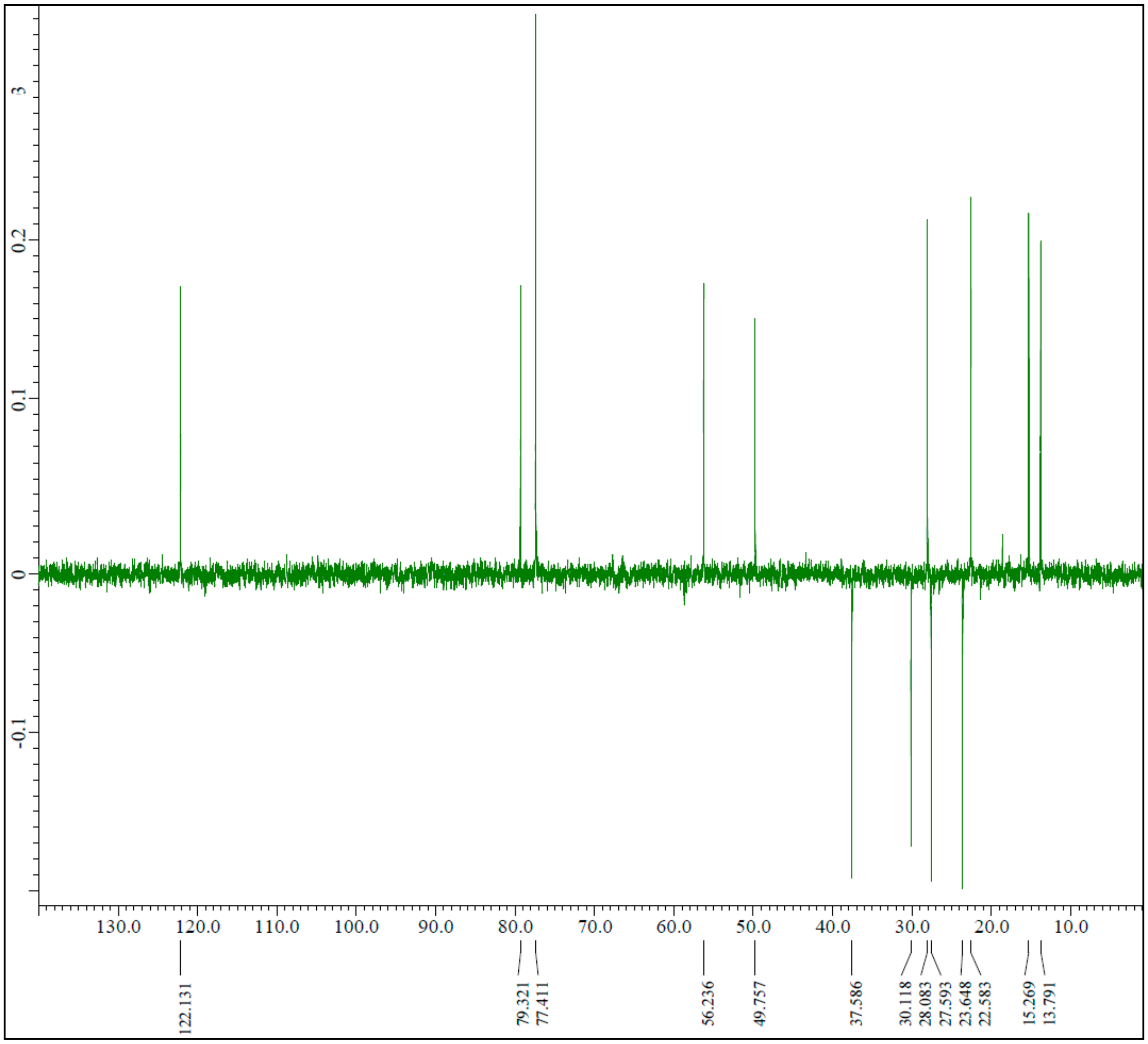

Figure S5: DEPT135 (125 MHz, $CDCl_3$) spectrum of **1**.

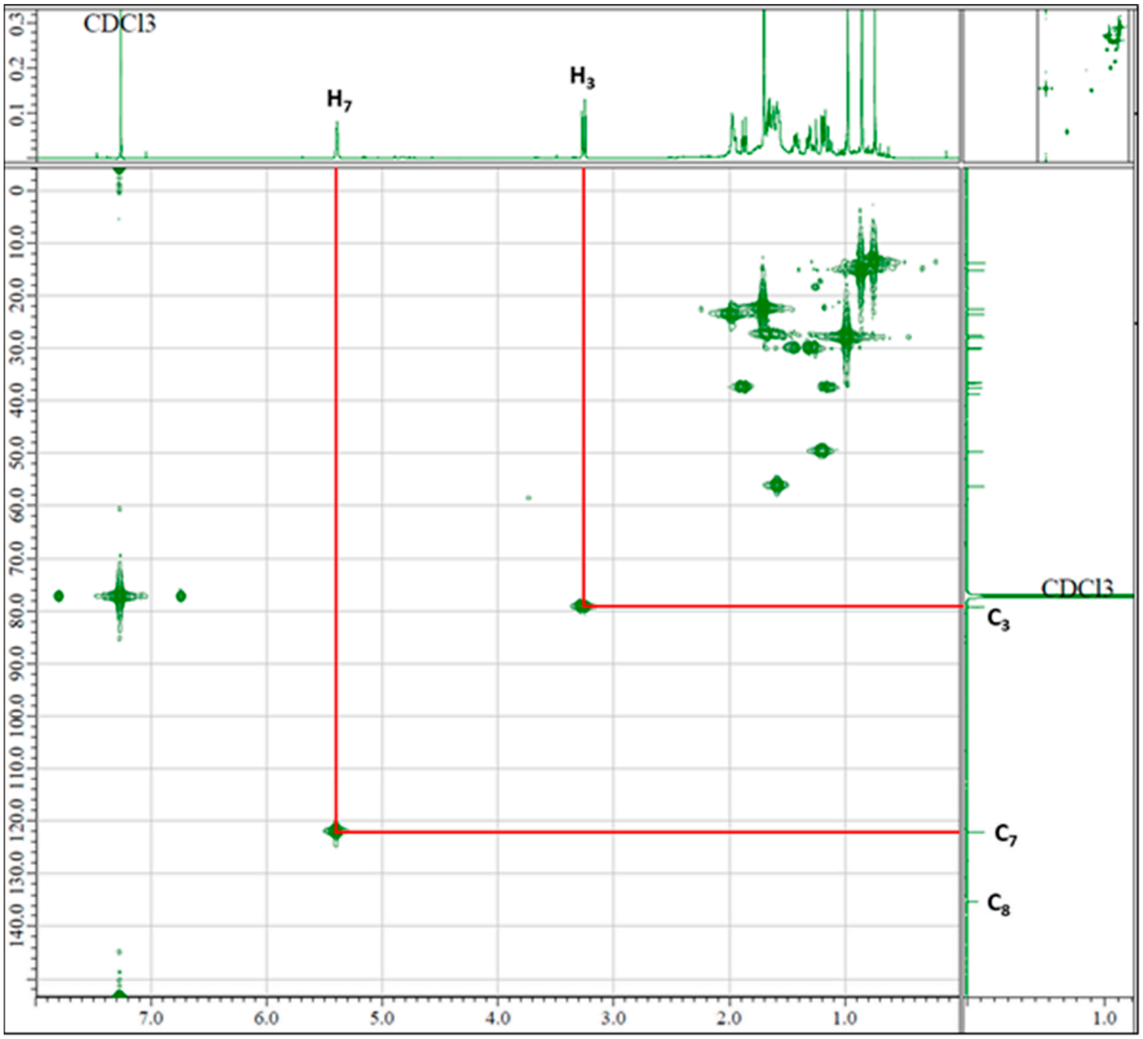

Figure S6: HMQC spectrum of **1**.

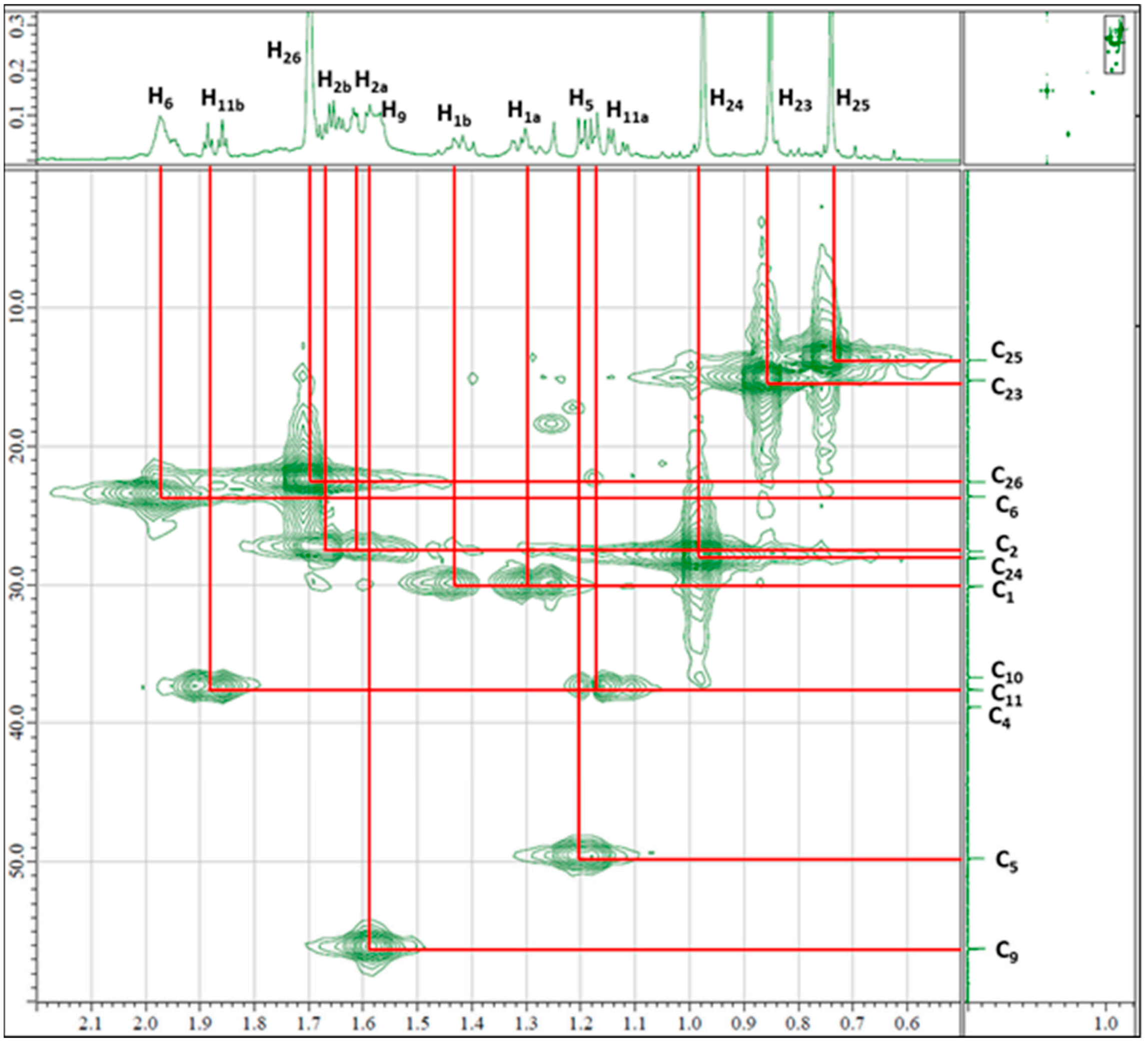

Figure S7: HMQC spectrum of **1** (From δ_C10 ppm to δ_C 60 ppm).

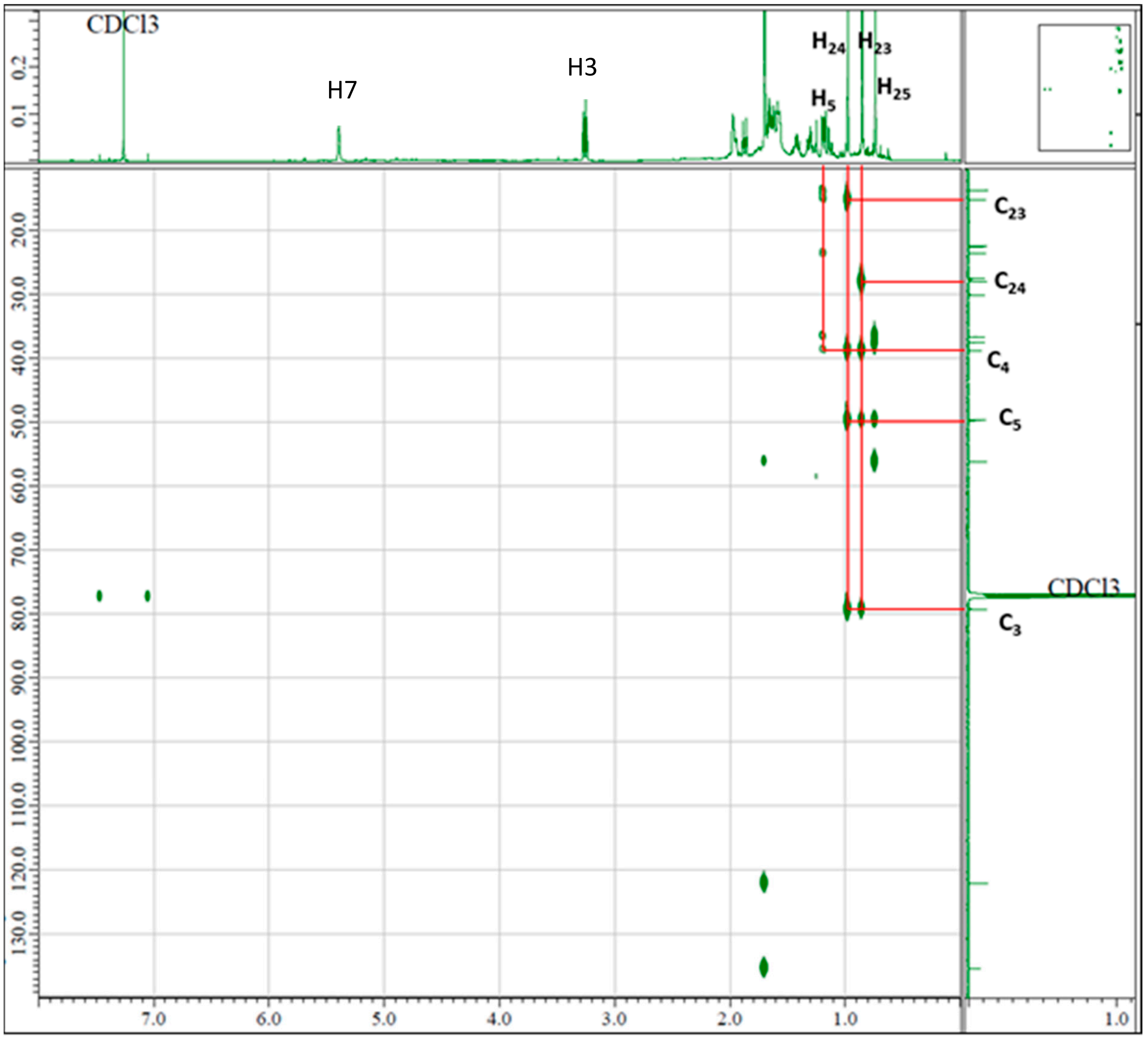

Figure S8: HMBC spectrum of **1**.

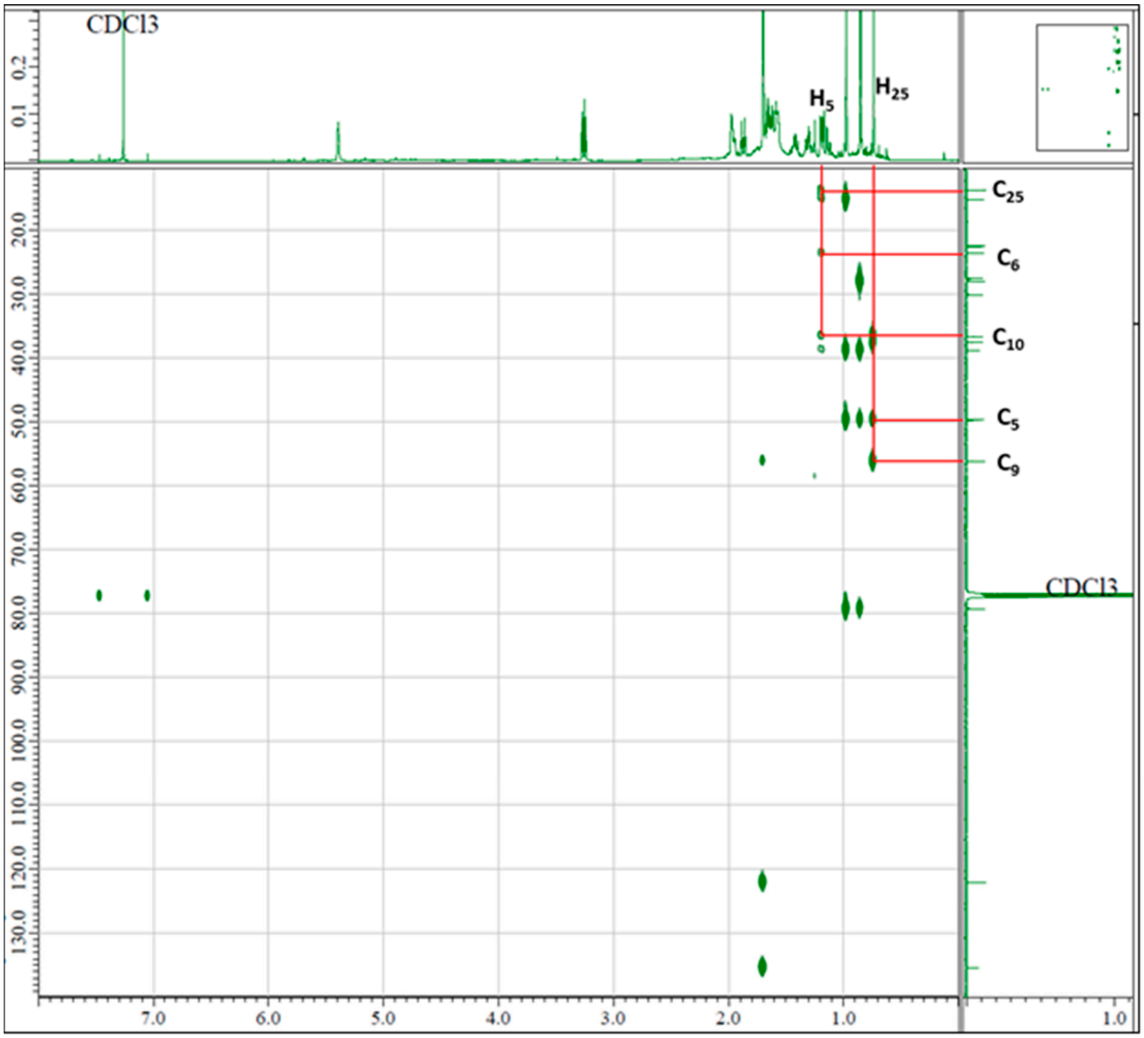

Figure S9: HMBC spectrum of **1** (From δ_C 10 ppm to δ_C 60 ppm).

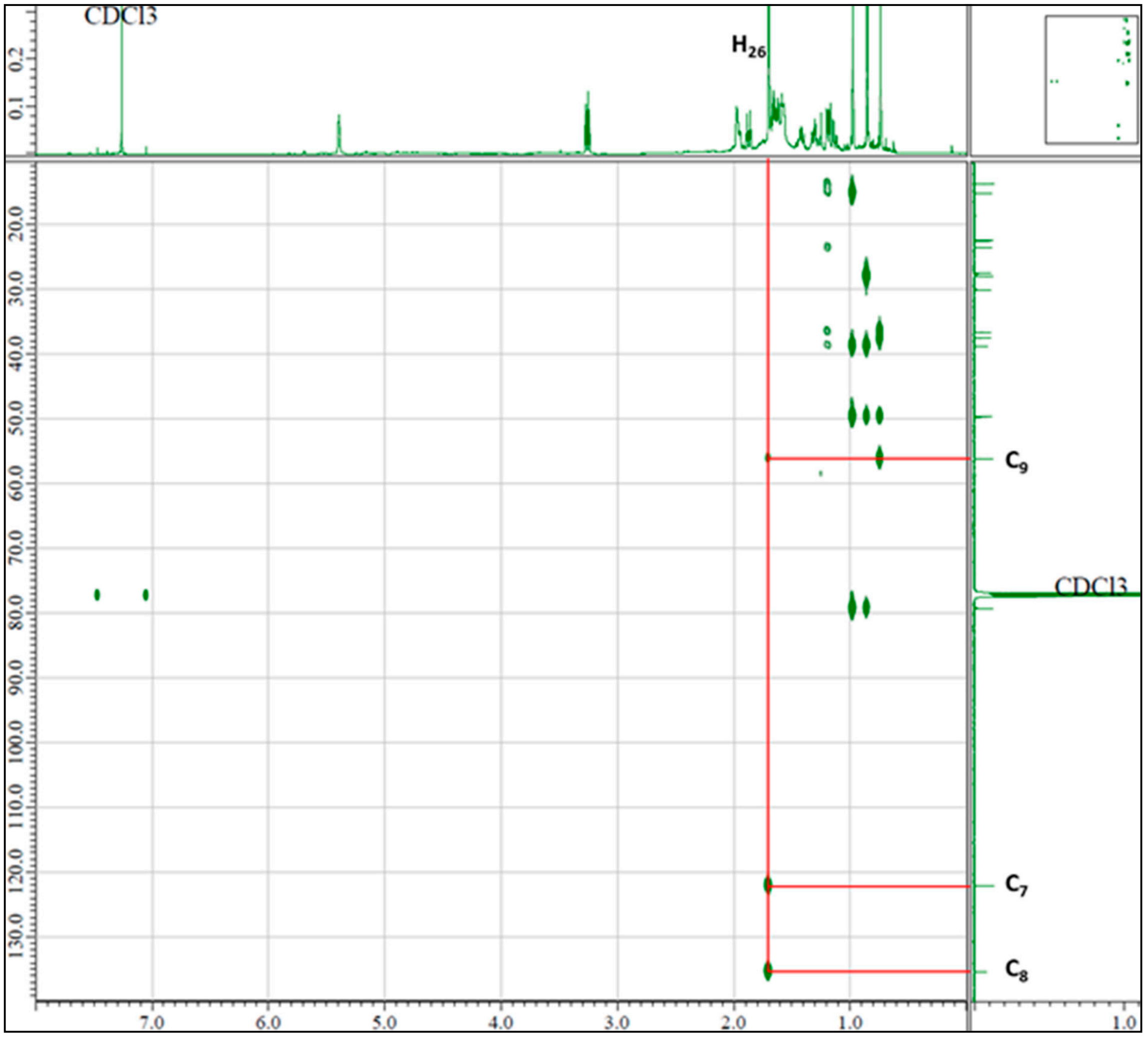

Figure S10: HMBC spectrum of **1** (From δ_C 10 ppm to δ_C 140 ppm).

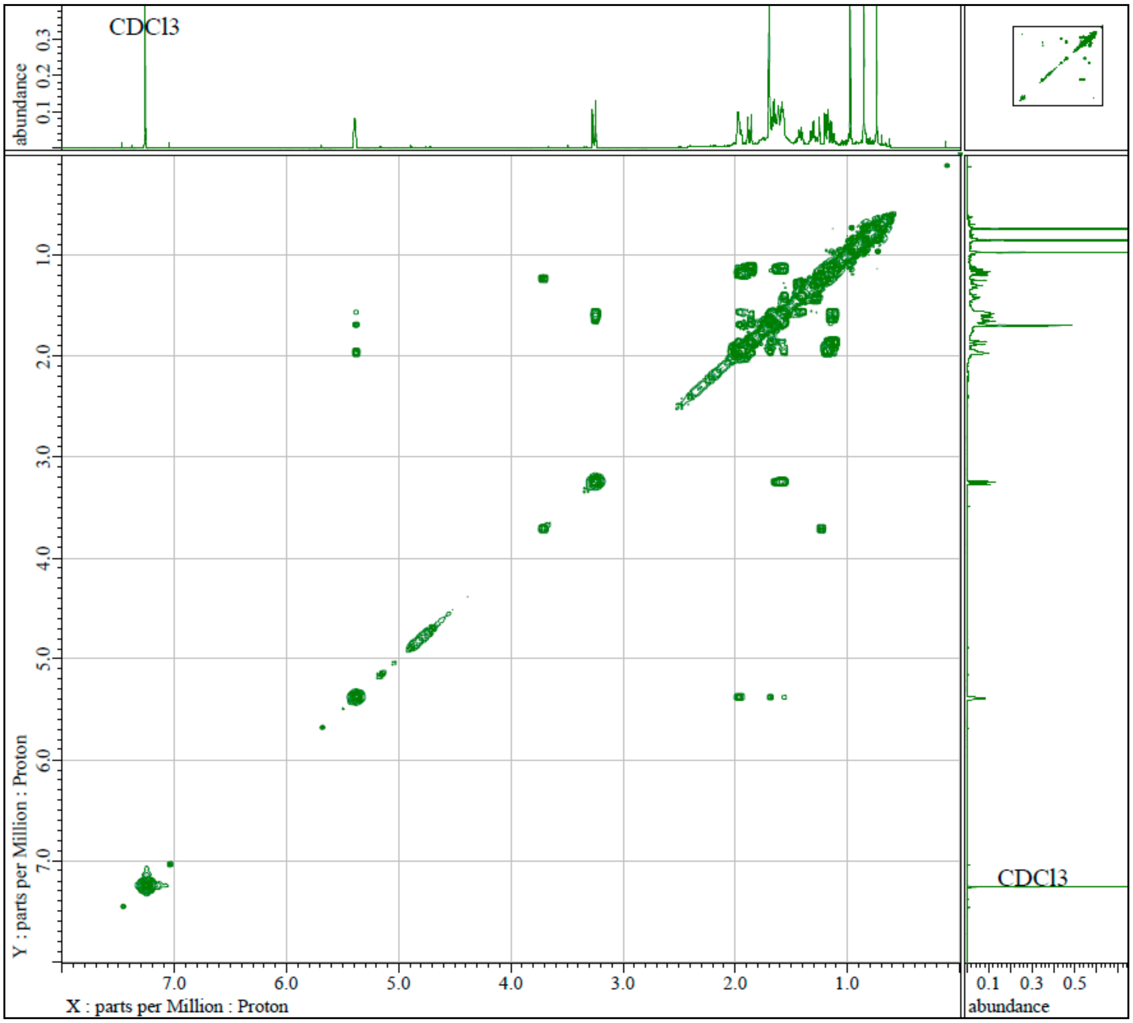

Figure S11: ^{1}H-^{1}H COSY spectrum of **1**.

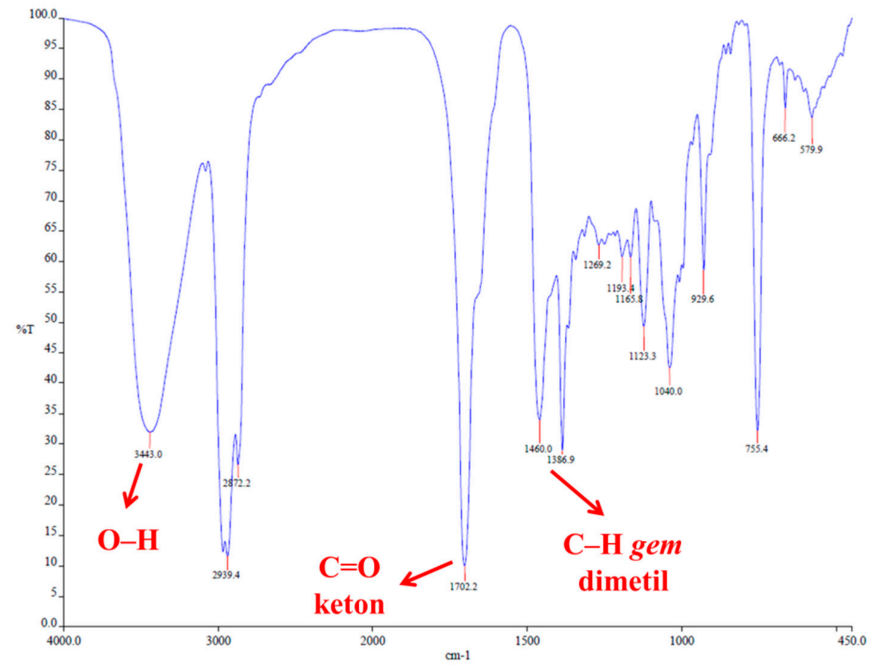

Figure 12. IR spectrum of 2

Single Mass Analysis
Tolerance = 10.0 mDa / DBE: min = -1.5, max = 50.0
Element prediction: Off
Number of isotope peaks used for i-FIT = 3

Monoisotopic Mass, Even Electron Ions
114 formula(e) evaluated with 1 results within limits (all results (up to 1000) for each mass)
Elements Used:
C: 0-500 H: 0-1000 O: 0-200
ZUL 03- 18 (0.323) Cm (18)
TOF MS ES-

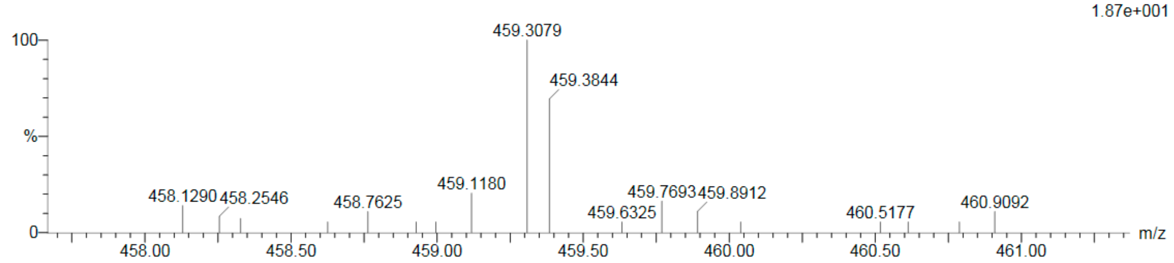

Minimum:				-1.5			
Maximum:		10.0	10.0	50.0			
Mass	Calc. Mass	mDa	PPM	DBE	i-FIT	i-FIT (Norm)	Formula
459.3844	459.3838	0.6	1.3	5.5	27.4	0.0	C30 H51 O3

Figure S13: HR-ESI-MS spectrum of **2**.

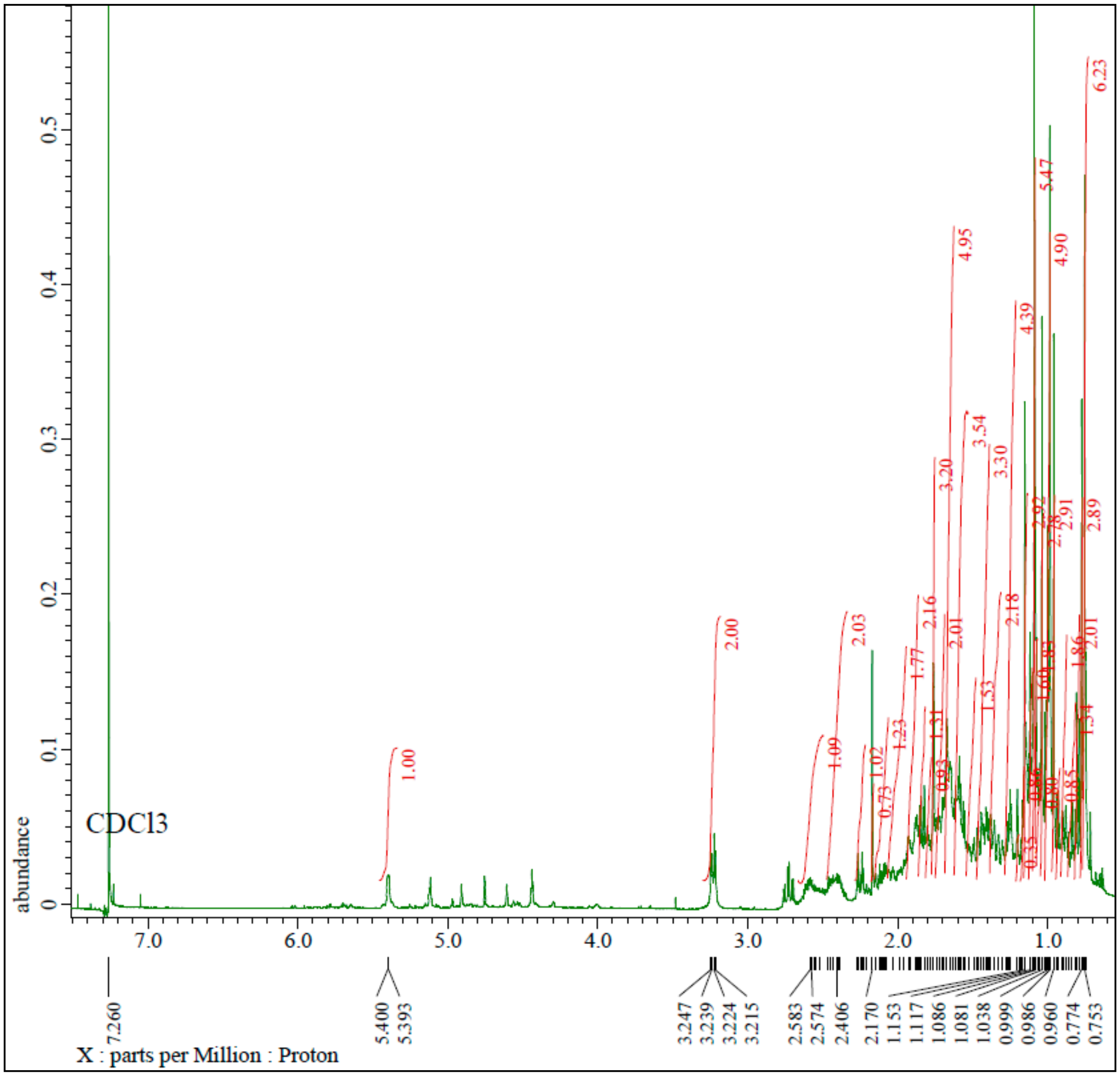

Figure S14: ^{1}H-NMR (500 MHz, $CDCl_3$) spectrum of **2**.

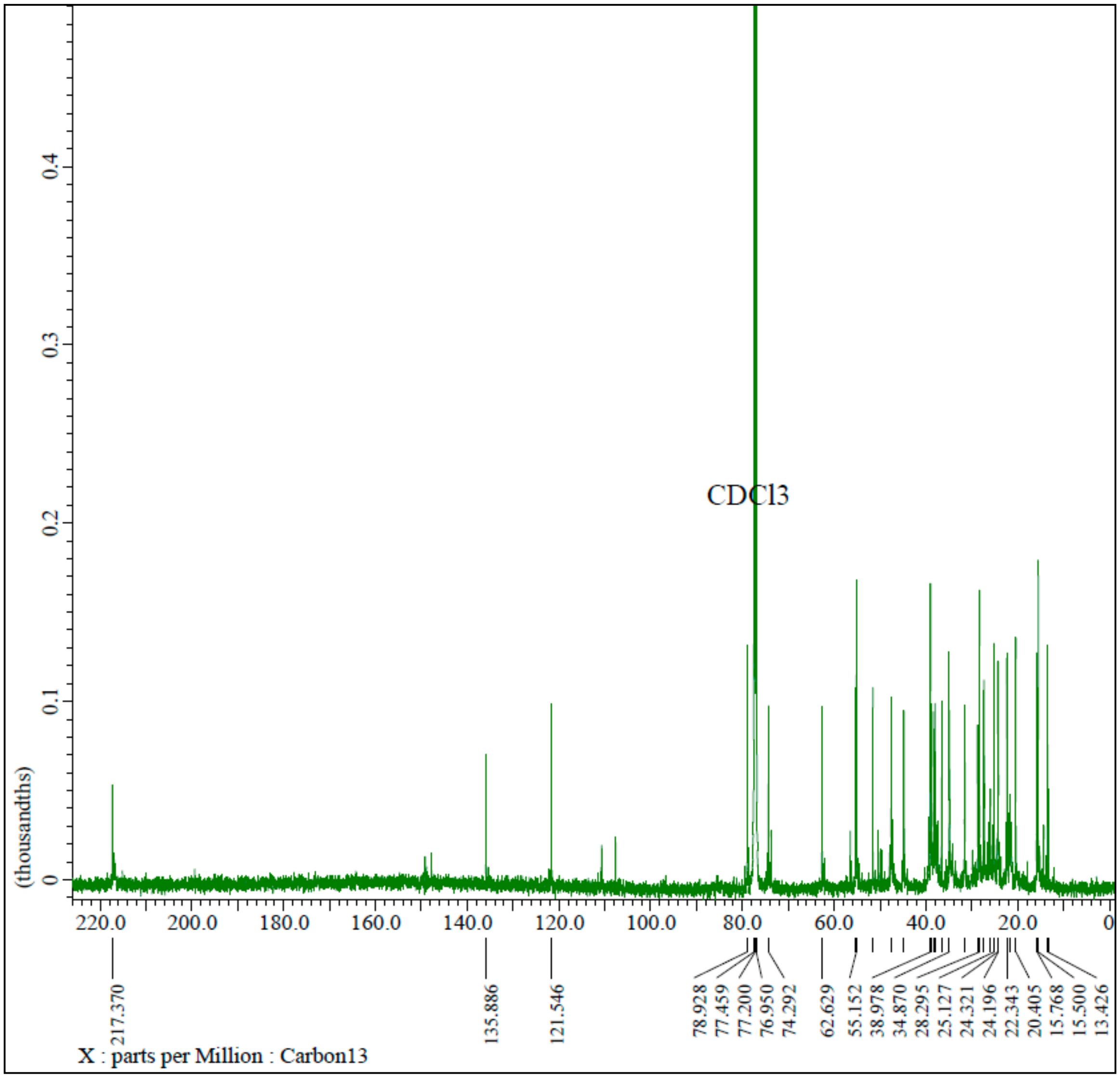

Figure S15: ^{13}C-NMR (125 MHz, $CDCl_3$) spectrum of **2**.

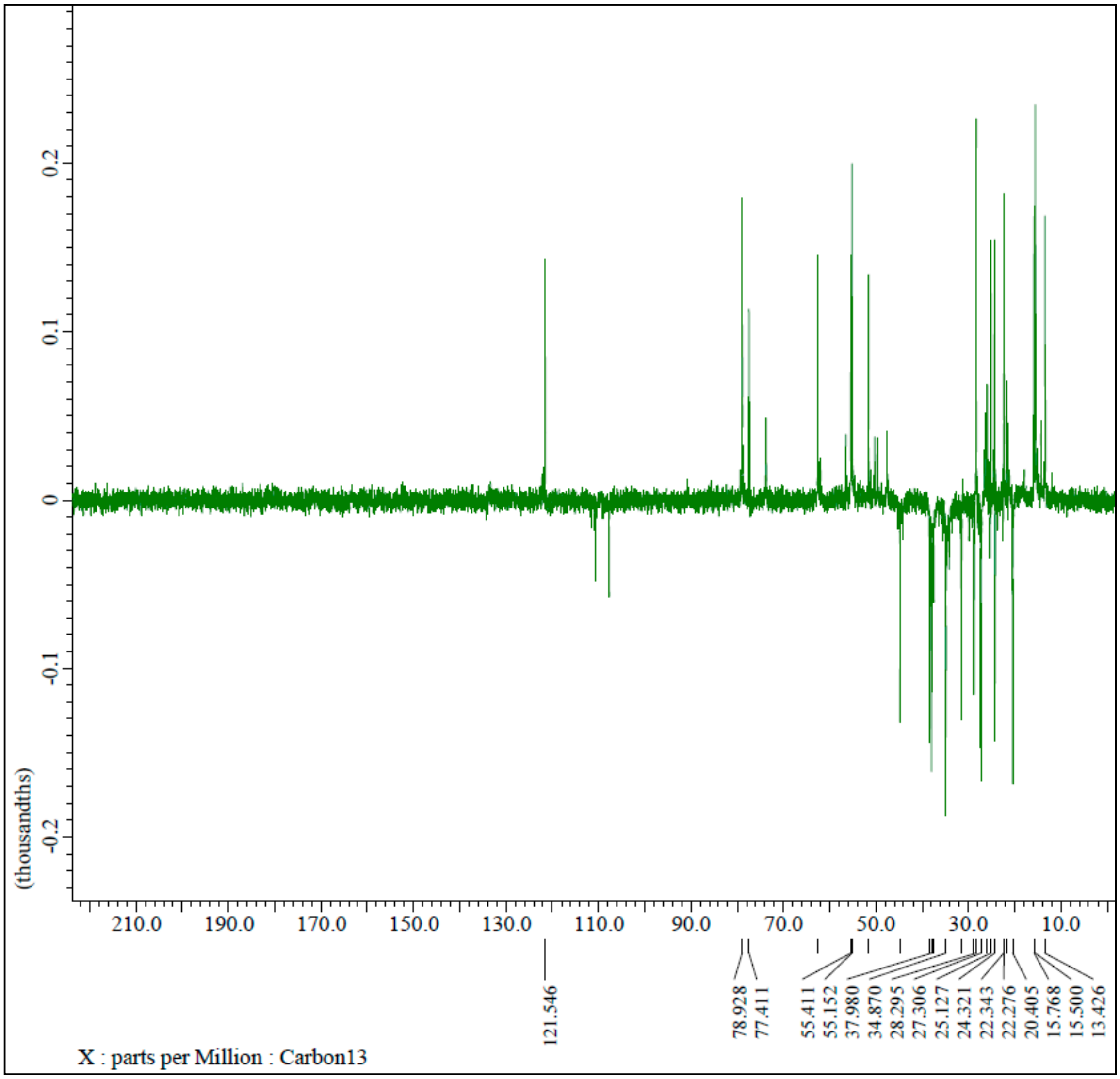

Figure S16: DEPT135 (125 MHz, $CDCl_3$) spectrum of **2**.

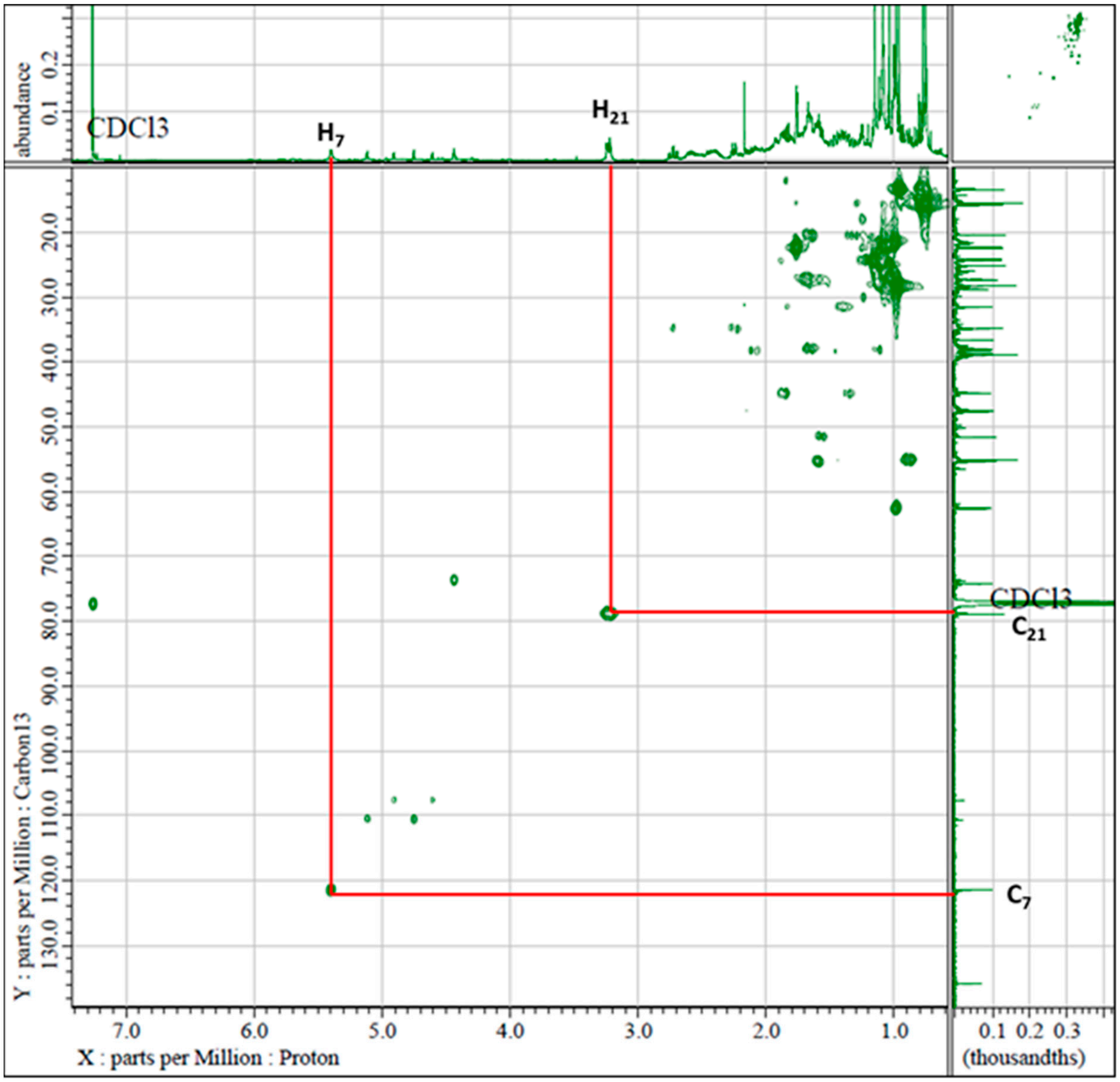

Figure S17: HMQC spectrum of **2**.

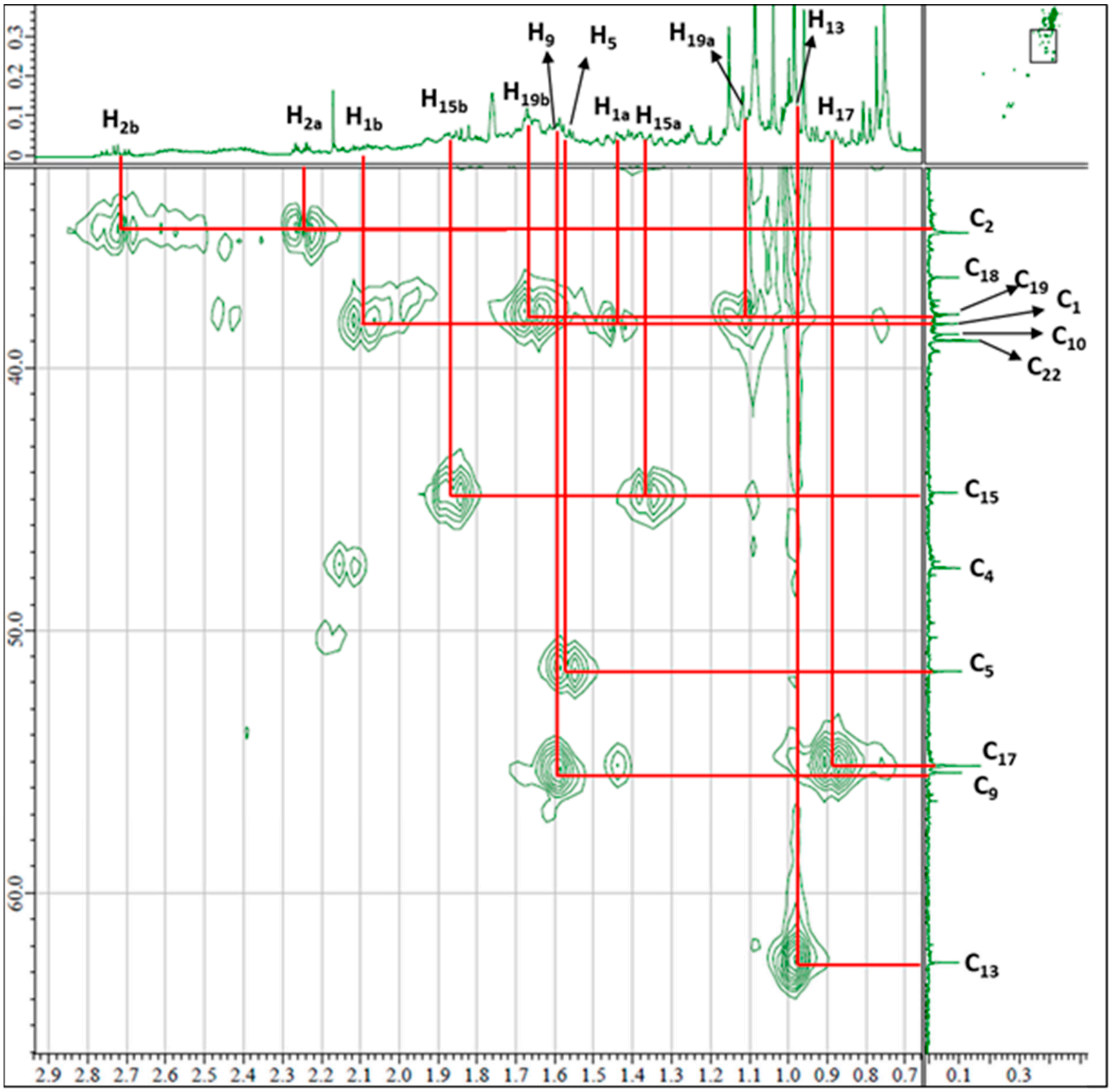

Figure S18: HMQC spectrum of **2** (From δ_C 30 ppm to 70 ppm).

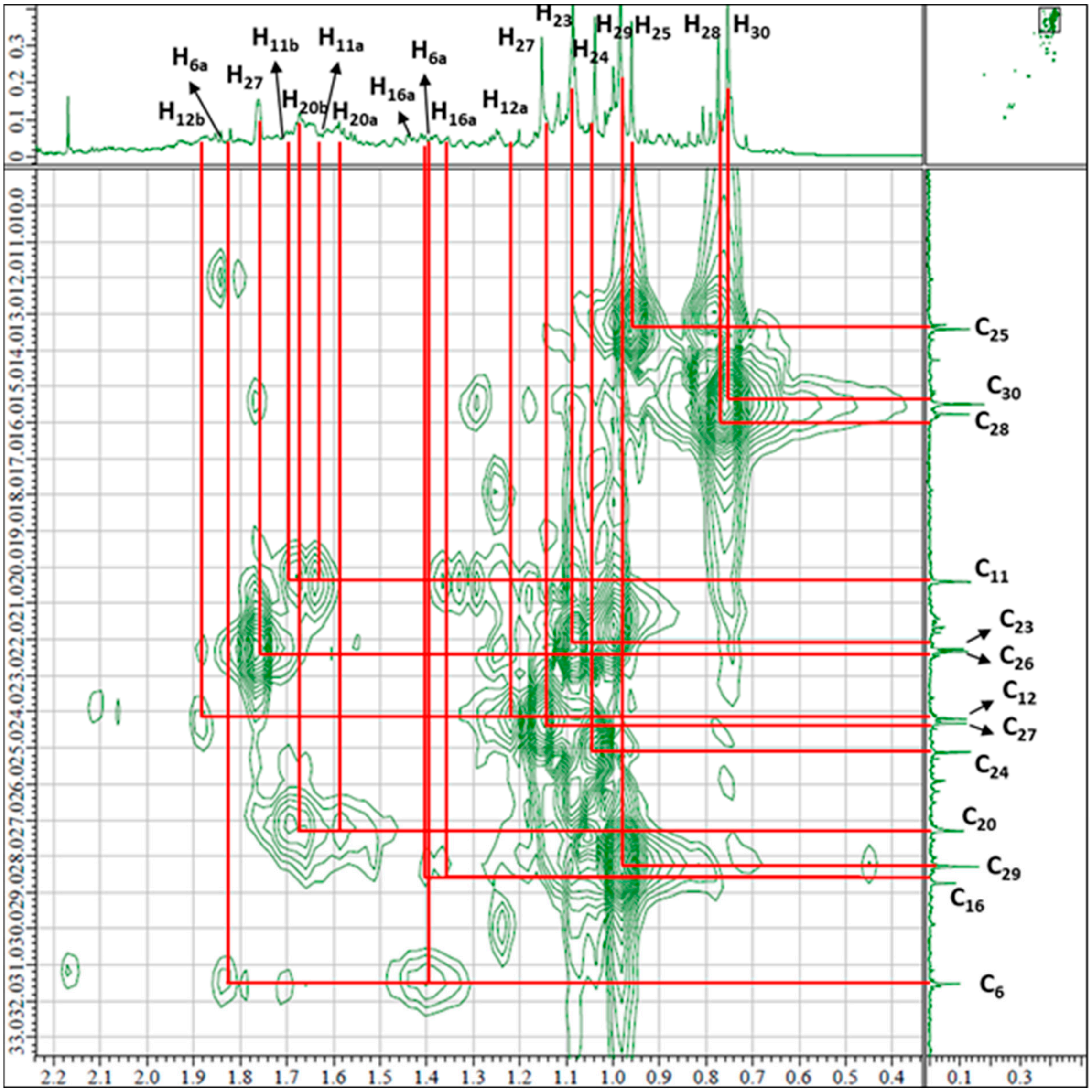

Figure S19: HMQC spectrum of **2** (From δ_C 13 ppm to 32 ppm).

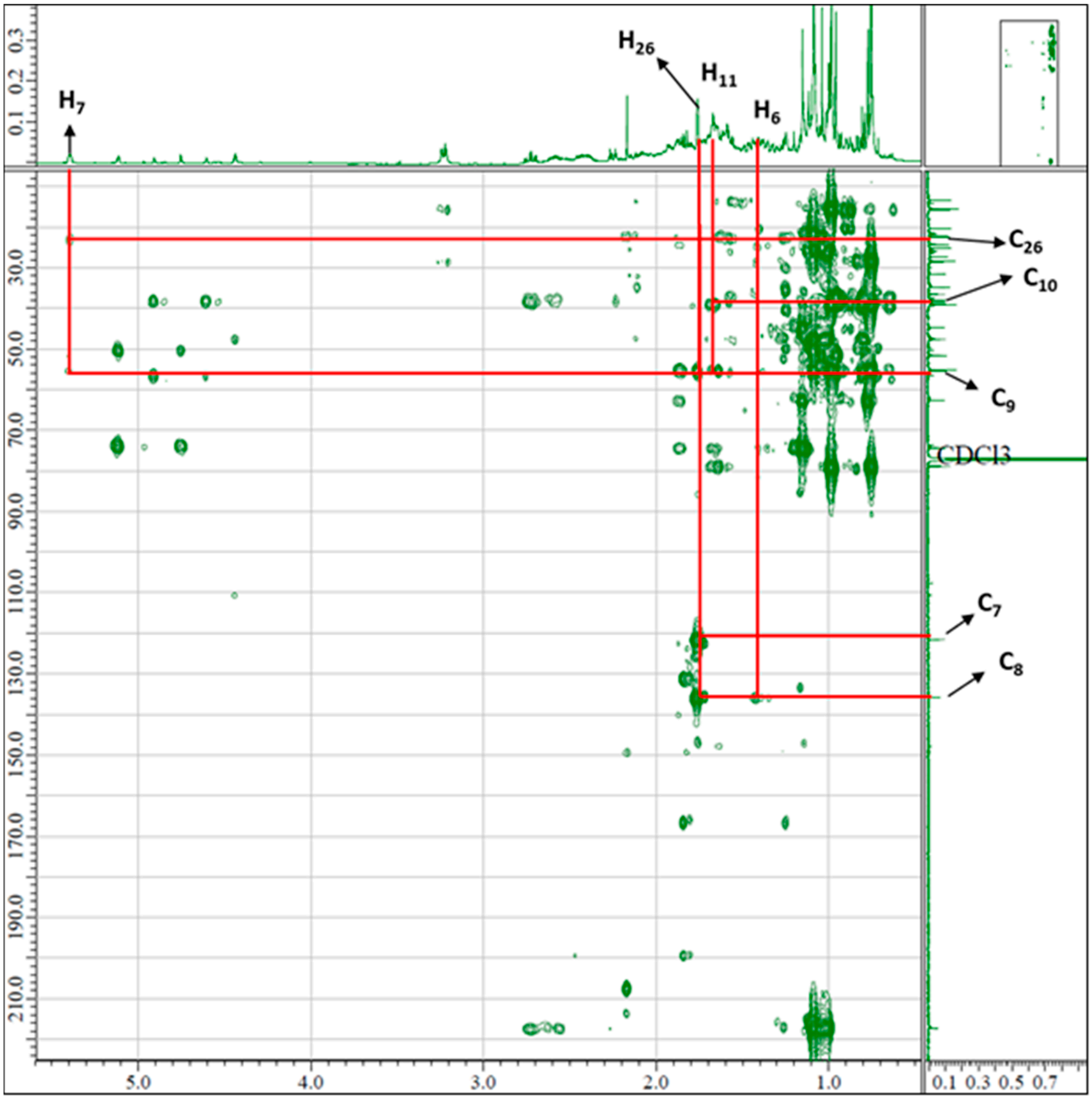

Figure S20: HMBC spectrum of **2**.

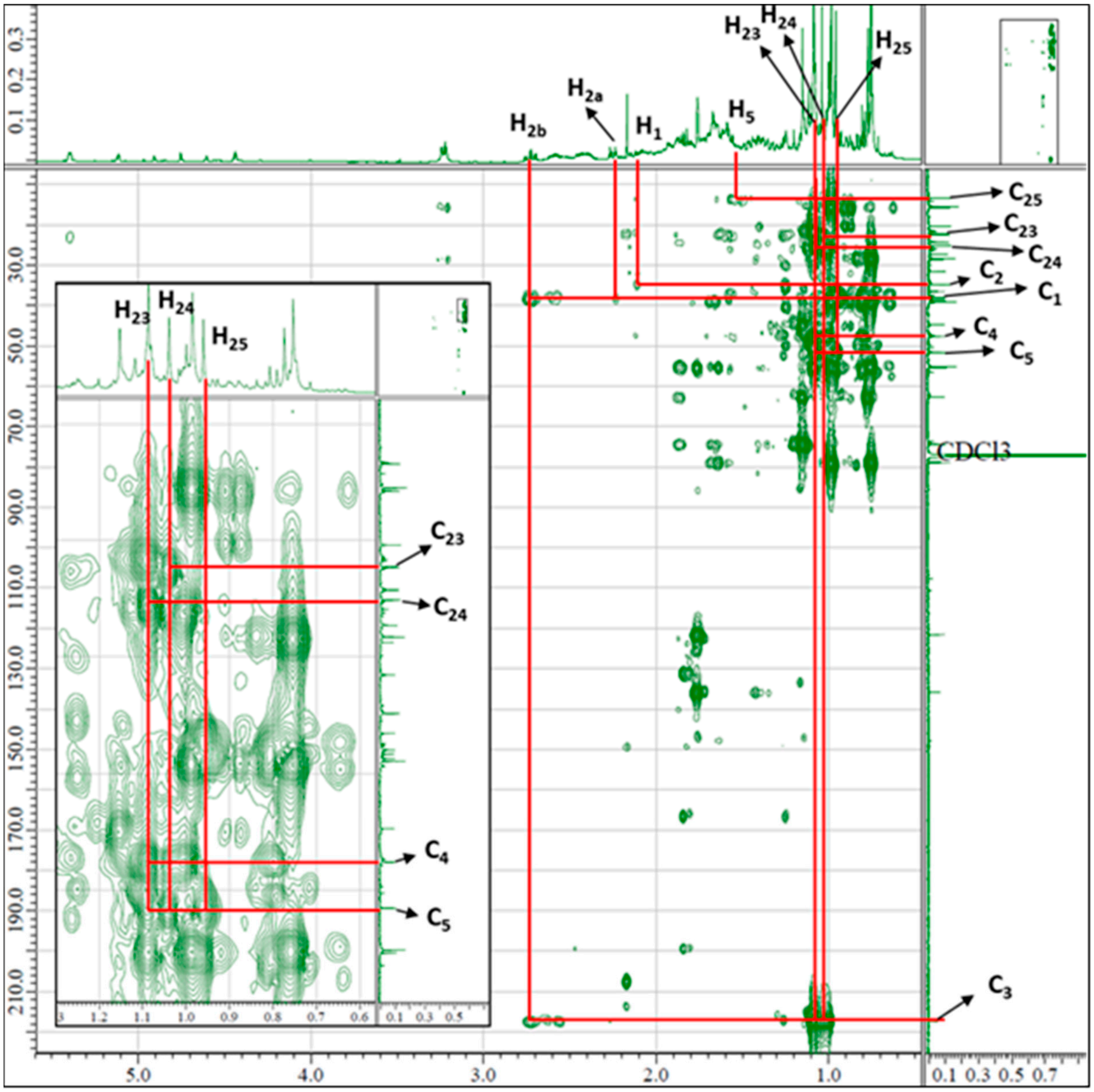

Figure S21: HMBC spectrum of **2** (expansion).

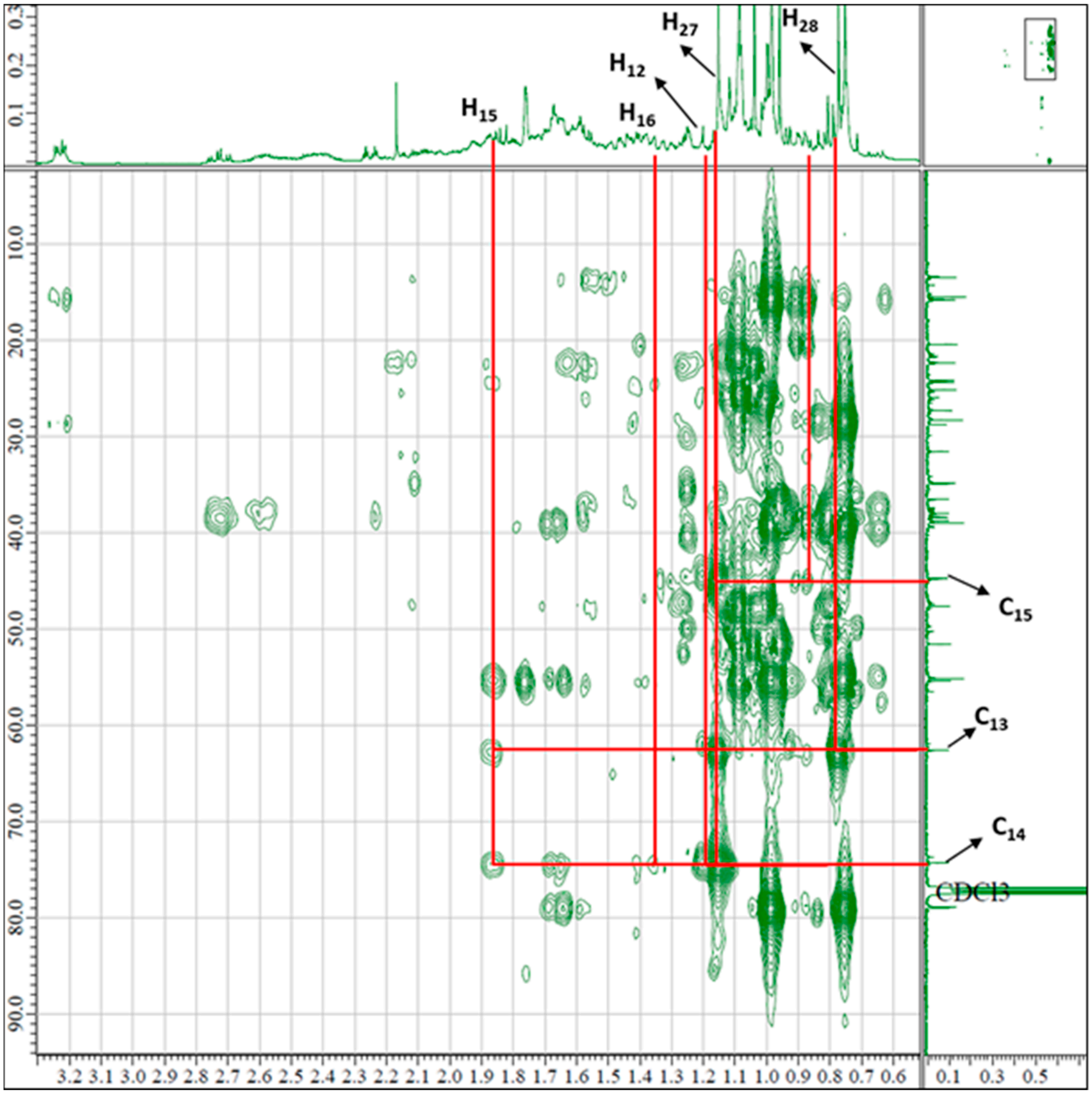

Figure S22: HMBC spectrum of **2** (From δ_C 40 ppm to 80 ppm).

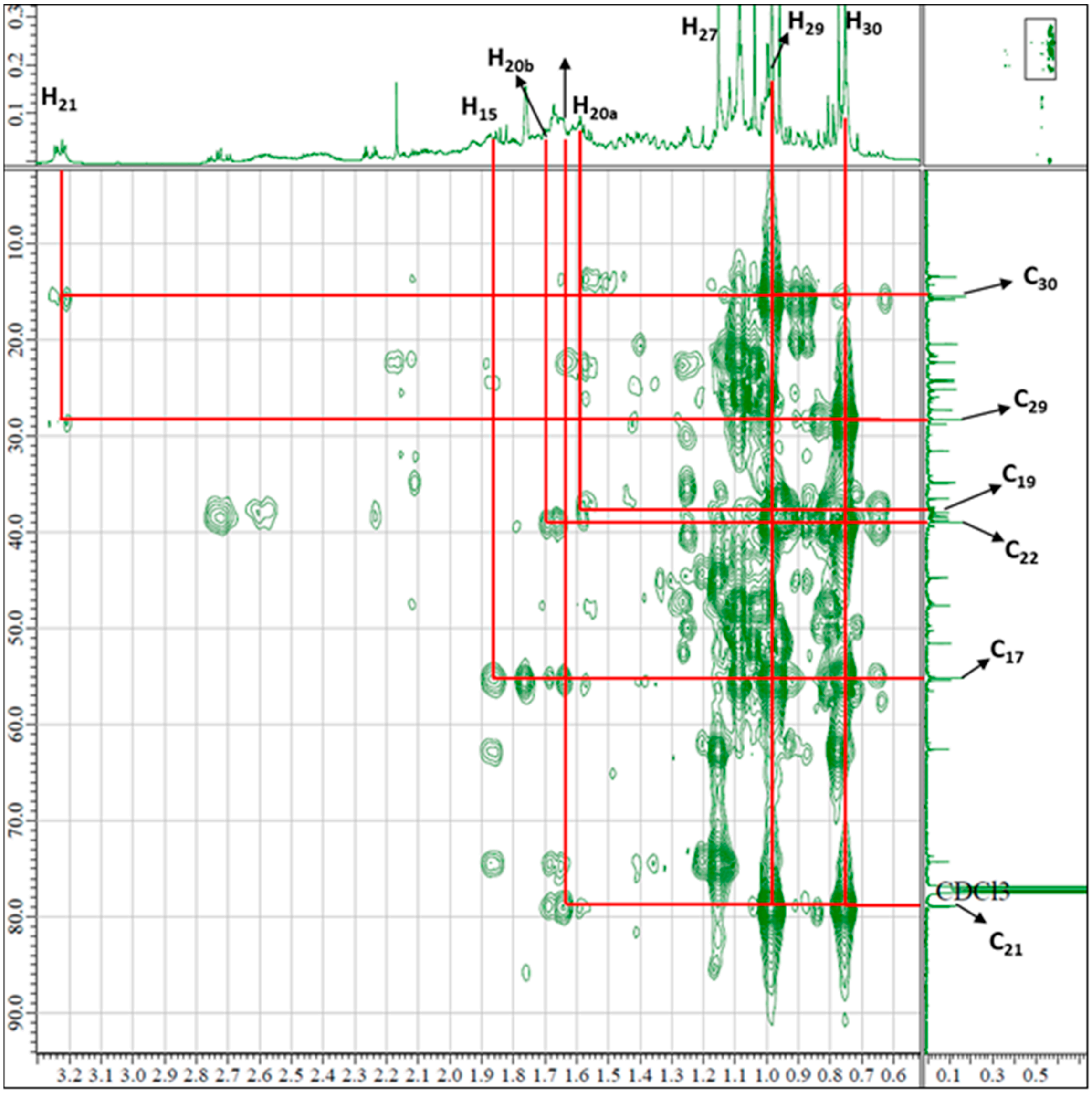

Figure S23: HMBC spectrum of **2** (From δ_C 10 ppm to 80 ppm).

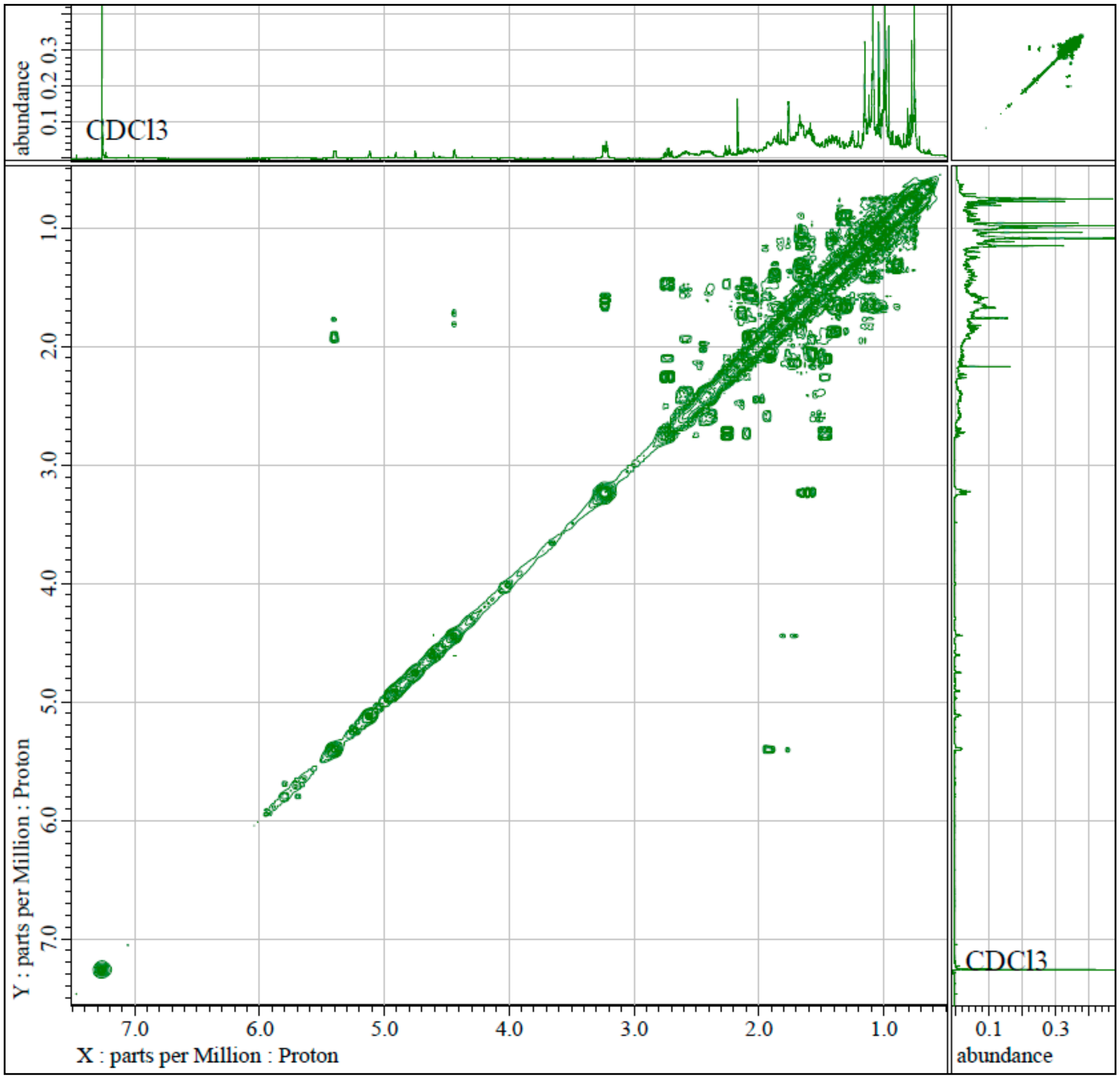

Figure S24: ^{1}H-^{1}H COSY spectrum of **2**.

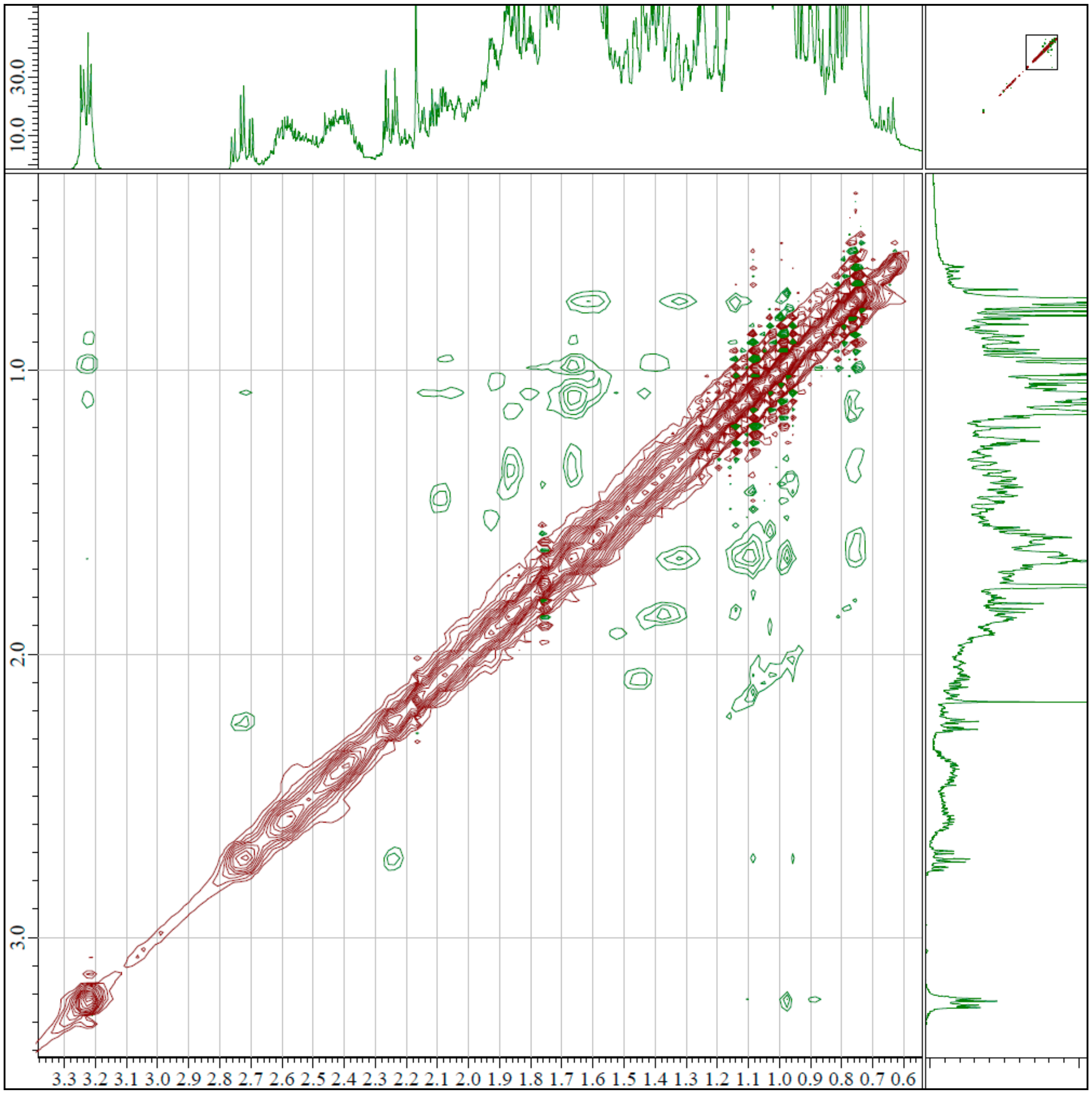

Figure S25: NOESY spectrum of **2**.

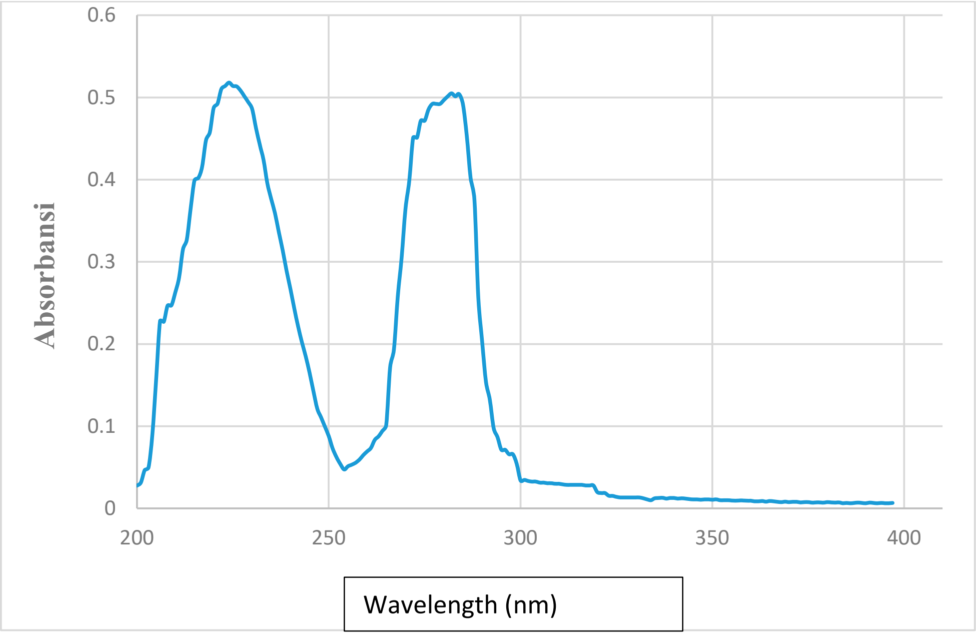

Figure 26. UV spectrum of **3**

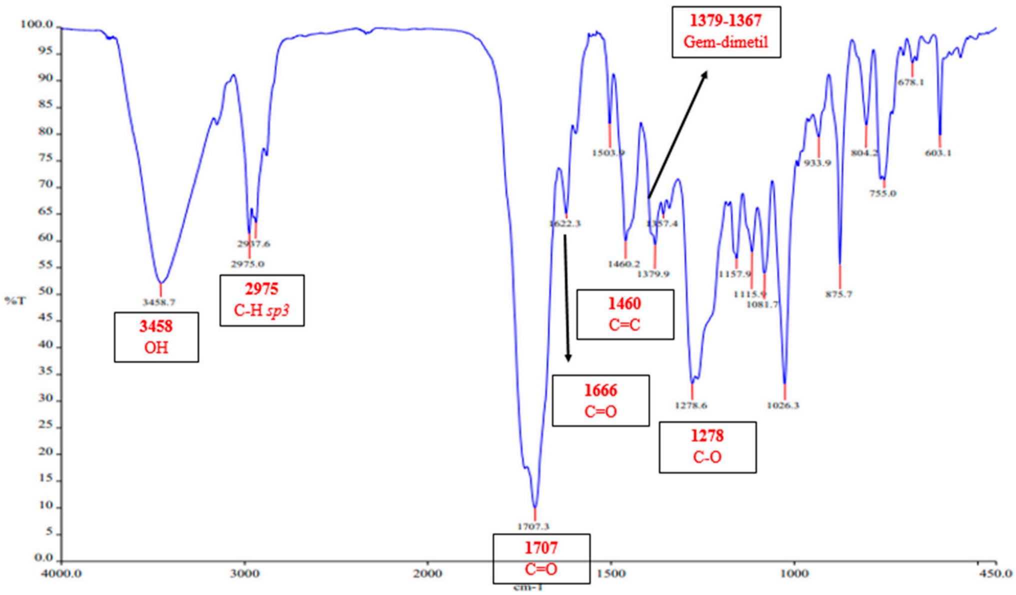

Figure 27. IR spectrum of **3**

Single Mass Analysis

Tolerance = 10.0 mDa / DBE: min = -1.5, max = 50.0
Element prediction: Off
Number of isotope peaks used for i-FIT = 3

Monoisotopic Mass, Even Electron Ions
127 formula(e) evaluated with 4 results within limits (up to 50 closest results for each mass)
Elements Used:
C: 0-500 H: 0-1000 O: 0-200
ISOLAT SARAH 1 31 (0.544) Cm (31:32)
TOF MS ES+

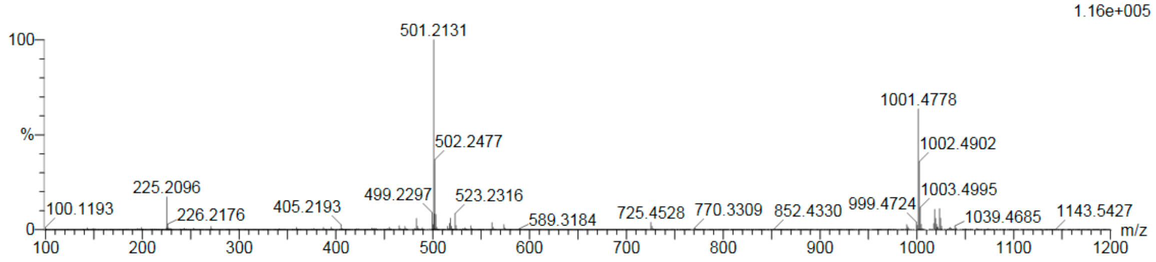

Minimum:				-1.5			
Maximum:		10.0	10.0	50.0			
Mass	Calc. Mass	mDa	PPM	DBE	i-FIT	i-FIT (Norm)	Formula
501.2131	501.2125	0.6	1.2	11.5	119.1	5.6	C27 H33 O9
	501.2183	-5.2	-10.4	2.5	122.4	8.9	C20 H37 O14
	501.2066	6.5	13.0	20.5	113.5	0.0	C34 H29 O4
	501.2218	-8.7	-17.4	24.5	118.6	5.1	C38 H29 O

Figure S28: HR-ESI-MS spectrum of **3.**

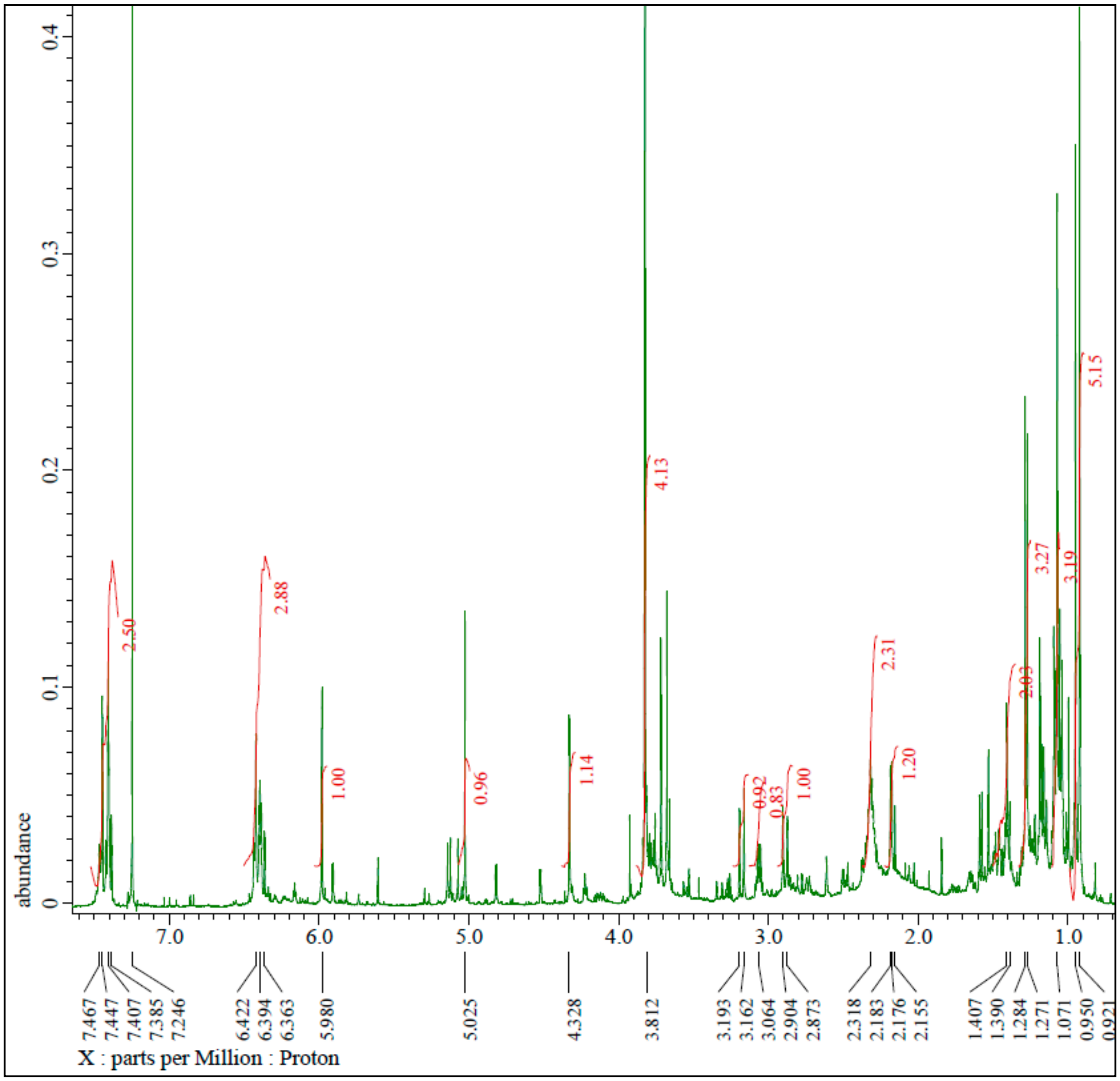

Figure S29: ^{1}H-NMR (500 MHz, $CDCl_3$) spectrum of **3**.

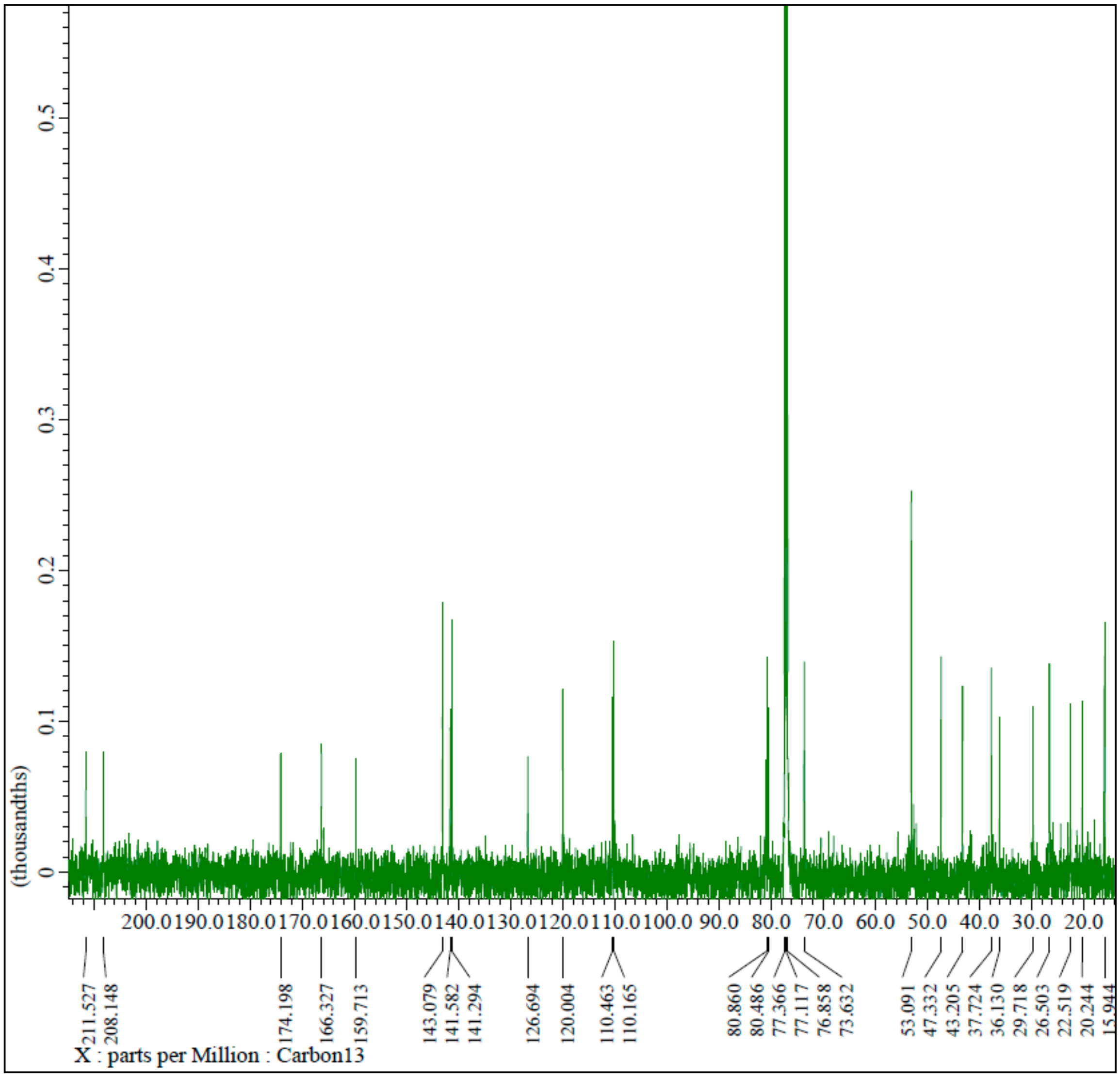

Figure S30: ^{13}C-NMR (125 MHz, $CDCl_3$) spectrum of **3**.

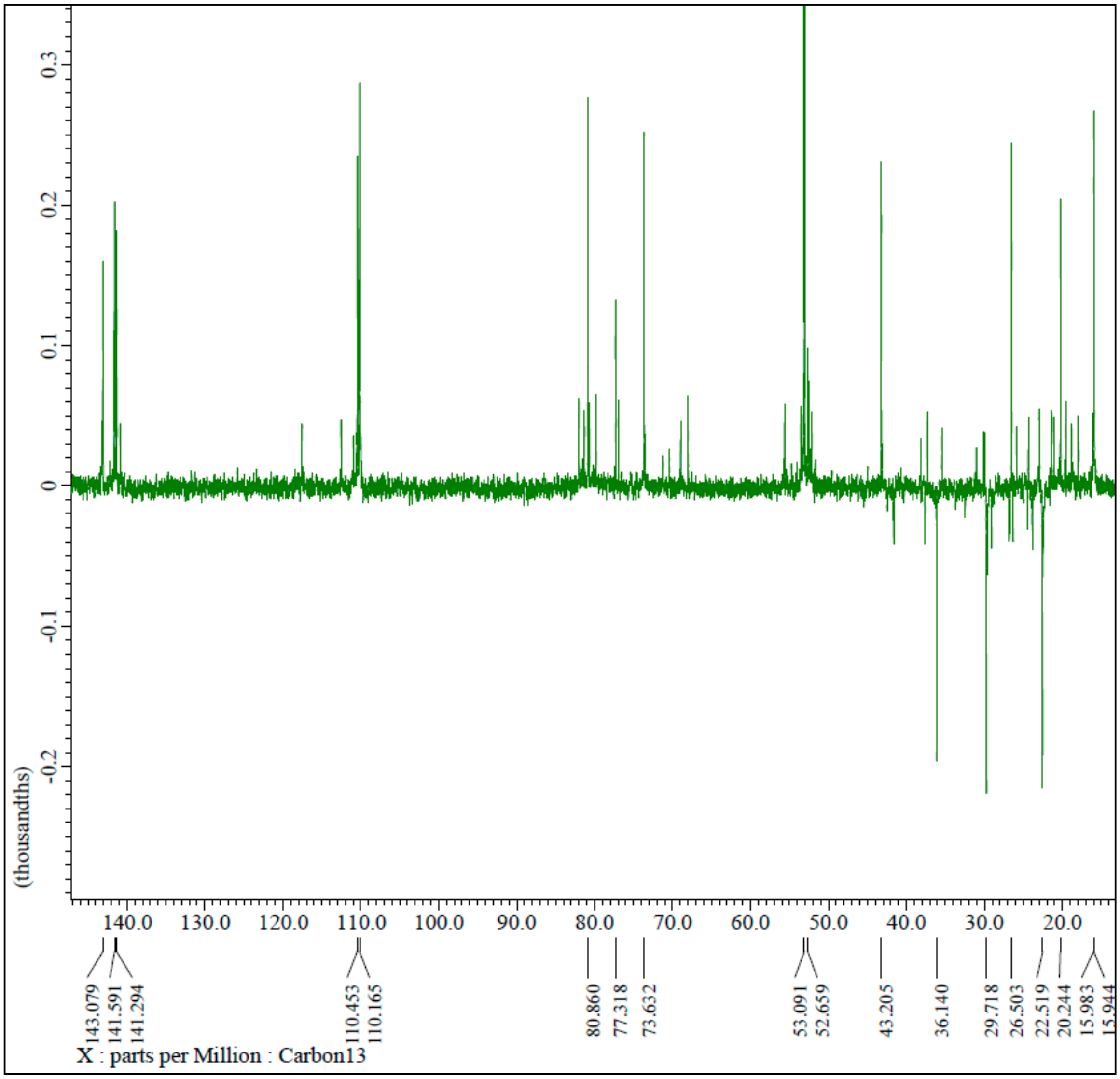

Figure S31: DEPT135 (125 MHz, $CDCl_3$) spectrum of **3**.

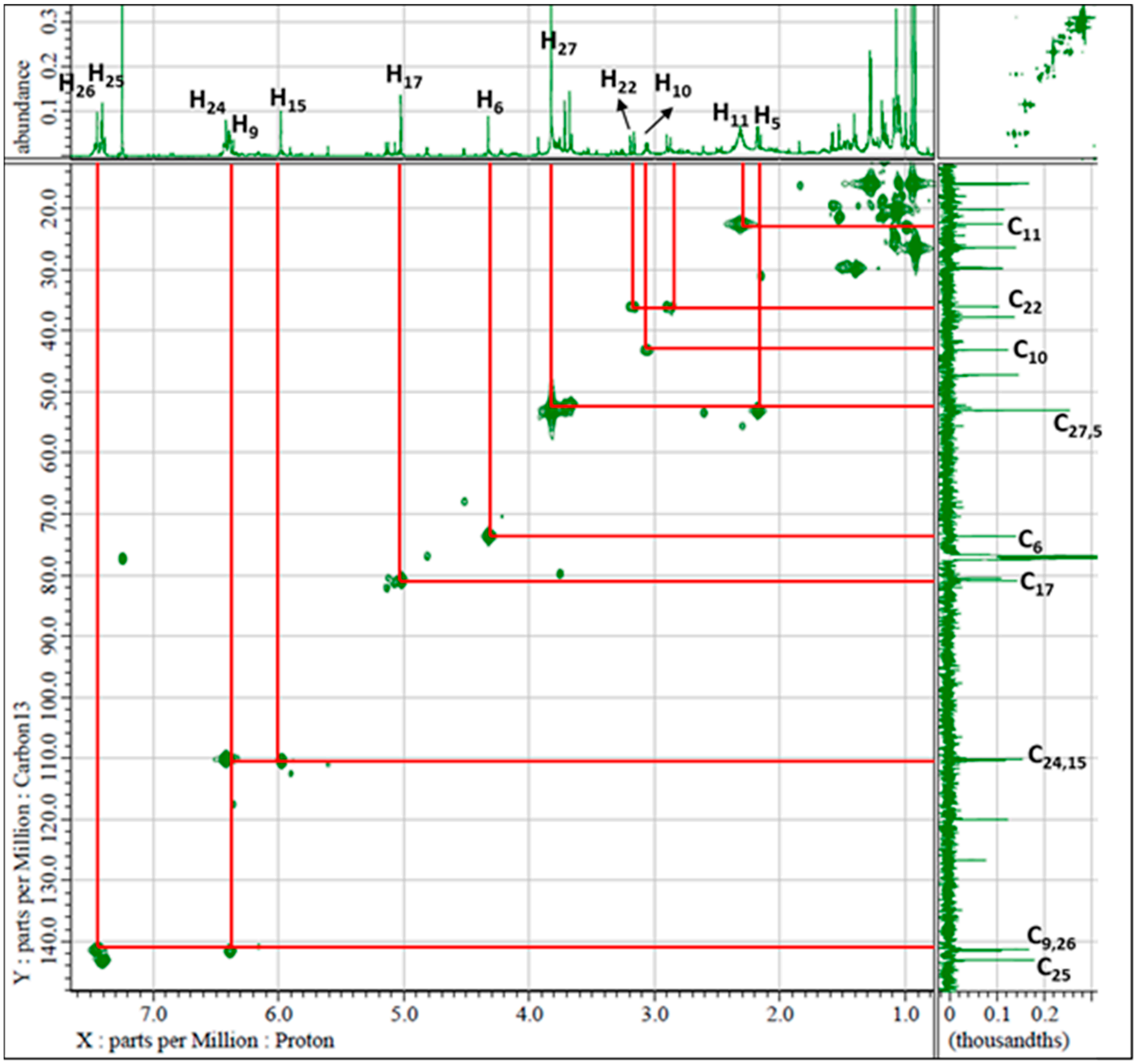

Figure S32: HMQC spectrum of **3**.

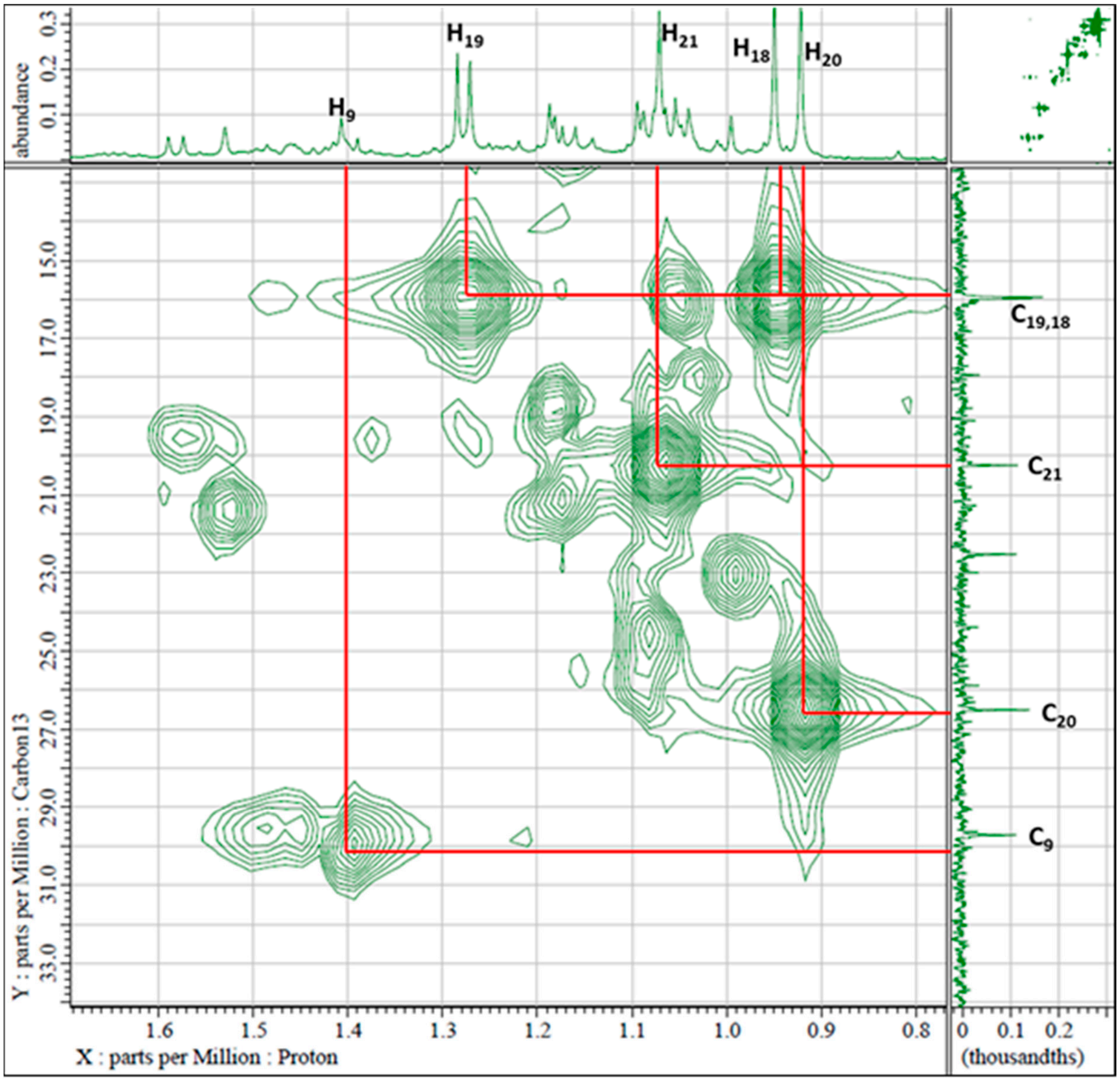

Figure S33: HMQC spectrum of **3** (From δ_C 15 ppm to 31 ppm).

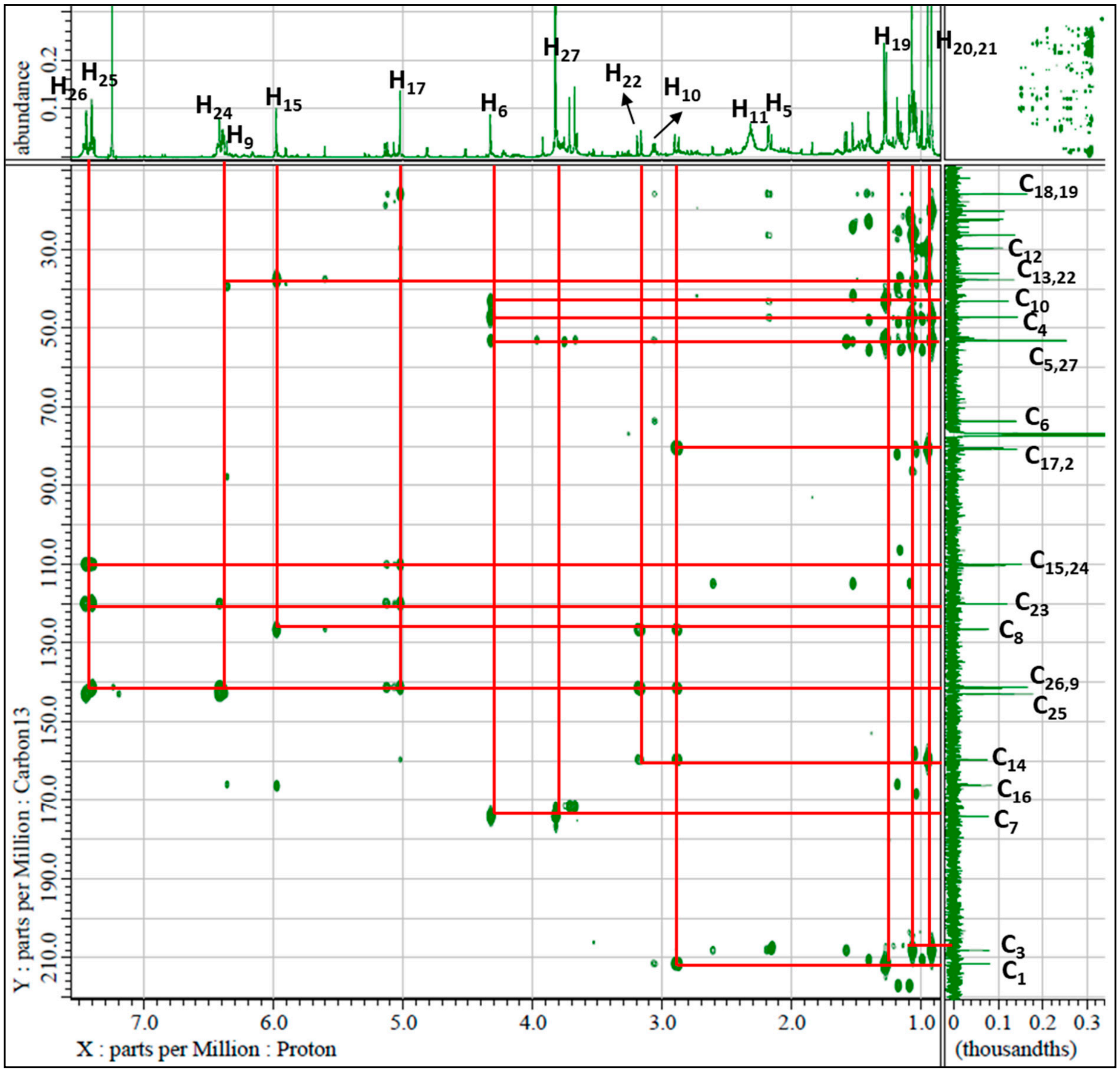

Figure S34: HMBC spectrum of **3**.

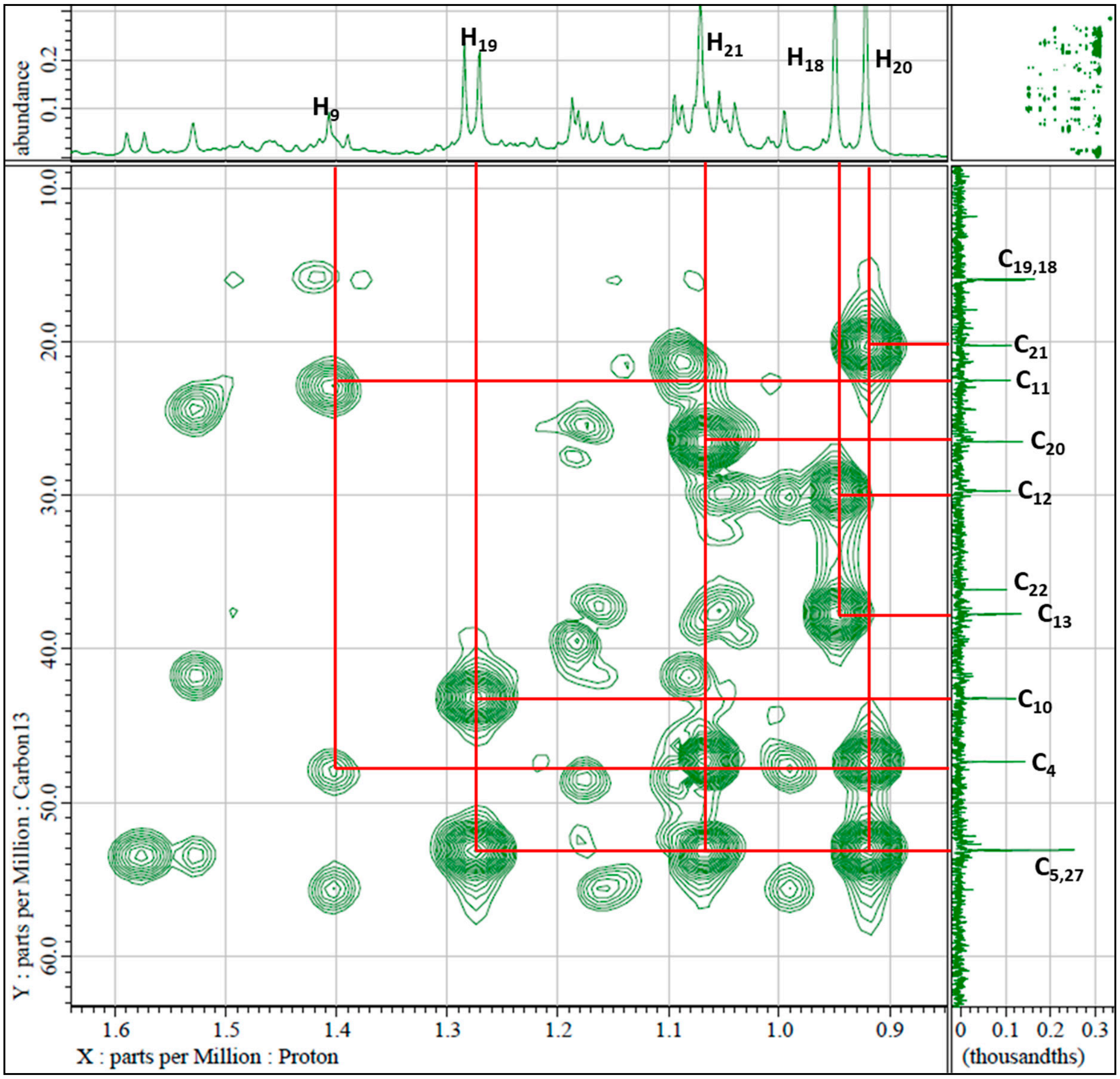

Figure S35: HMBC spectrum of **3** (From δ_C 10 ppm to 60 ppm).

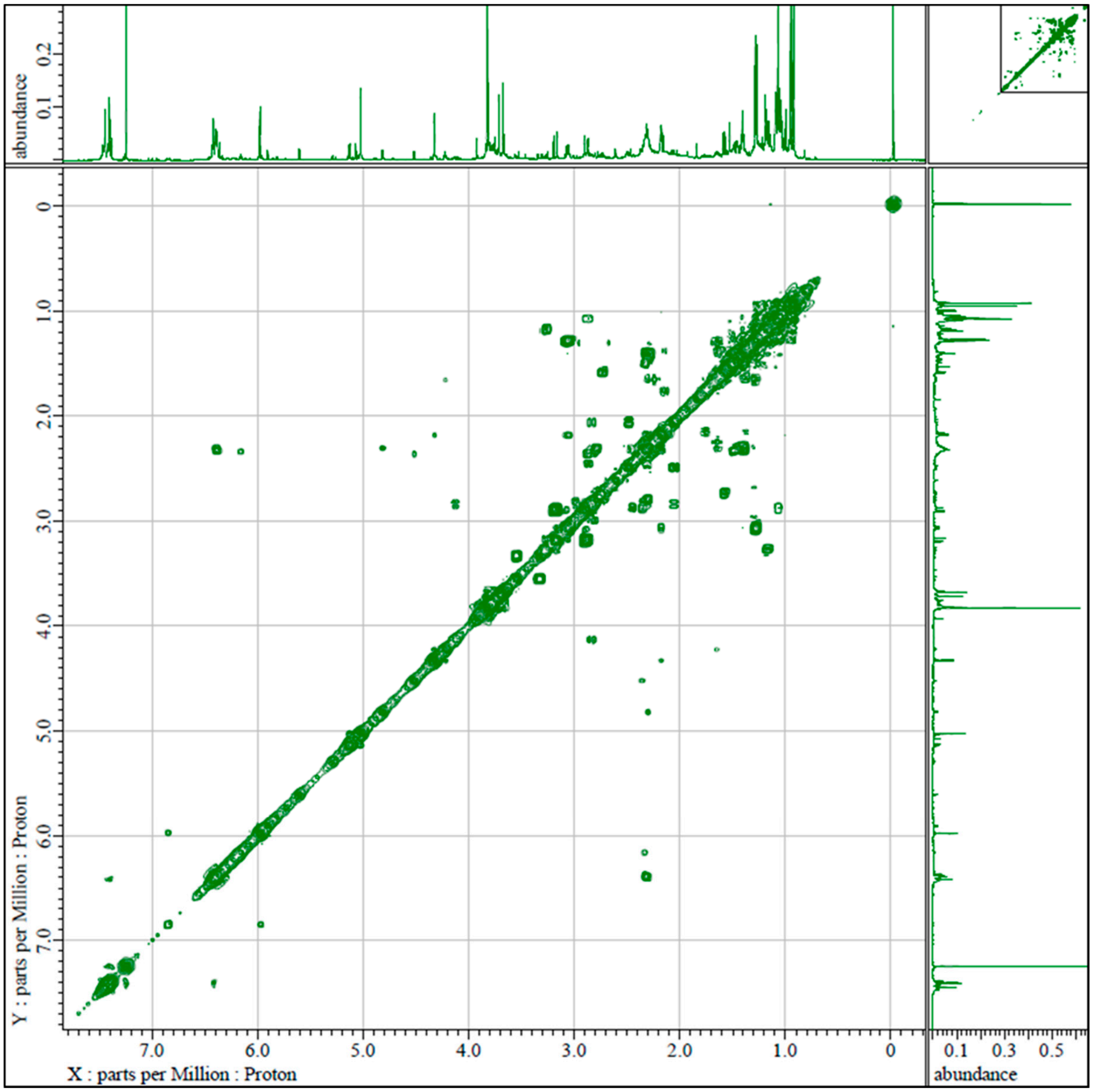

Figure S36: ^{1}H-^{1}H COSY spectrum of **3**.

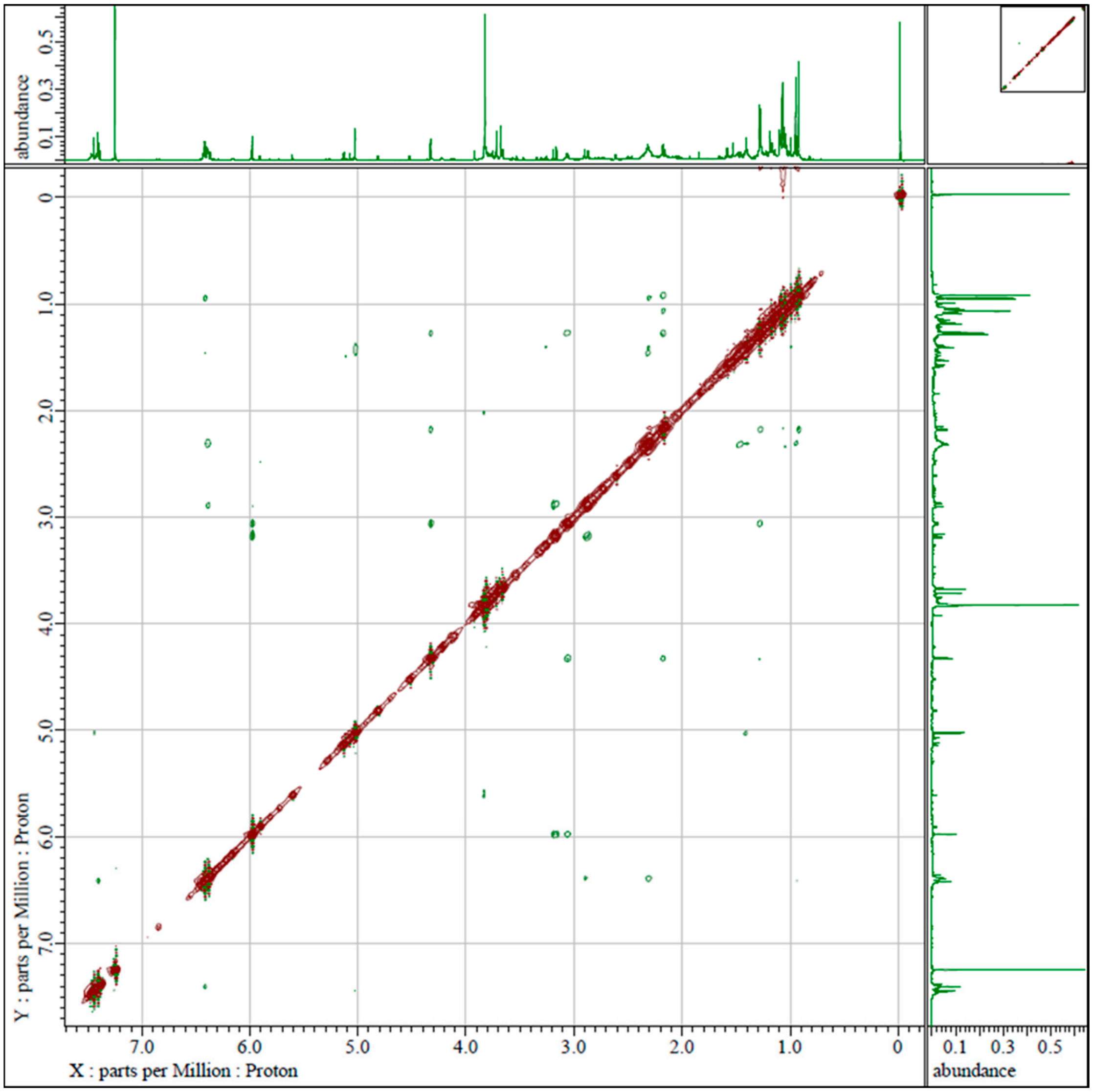

Figure S37: NOESY spectrum of **3**.

CAS SciFinder^n **Task History**

Initiating Search

October 11, 2022, 10:55AM

Substances:

Filtered By:

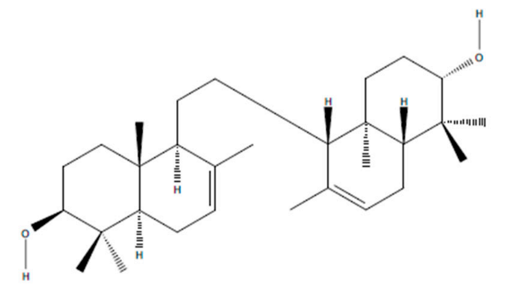

Structure Match: **As Drawn**

Search Tasks

Task	Search Type	View
Exported: Returned Substance Results + Filters	**Substances**	View Results

CAS SciFinder^n

Substances (0)

View in SciFinder^n

We couldn't find any results. Please update your search query and try again.

Substances with (0) results

Copyright © 2022 American Chemical Society (ACS). All Rights Reserved.
Internal use only. Redistribution is subject to the terms of your SciFinder^n License Agreement and CAS information Use Policies.

Figure S38: Scifinder reports of compounds **1**.

CAS SciFinderⁿ

Substances (8)

View in SciFinderⁿ

1 Similarity Score: **98**

90686-40-1

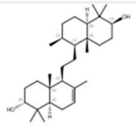

Absolute stereochemistry shown

$C_{30}H_{52}O_2$

2-Naphthalenol, decahydro-1,1,4a,6-tetramethyl-5-[2-[(1*S*,4a*R*,6*R*,8a*R*)-1,4,4a,5,6,7,8,8a-octahydro-6-hydroxy-2,5,5,8a-tetramethyl-1-naphthalenyl]ethyl]-, (2*R*,4a*R*,5*S*,6*S*,8a*R*)-

6 References | 0 Reactions | 0 Suppliers

Key Physical Properties	Value	Condition
Molecular Weight	444.73	-
Boiling Point (Predicted)	522.2±50.0 °C	Press: 760 Torr
Density (Predicted)	0.962±0.06 g/cm^3	Temp: 20 °C; Press: 760 Torr
pKa (Predicted)	15.18±0.70	Most Acidic Temp: 25 °C

2 Similarity Score: **98**

90686-41-2

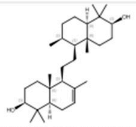

Absolute stereochemistry shown

$C_{30}H_{52}O_2$

2-Naphthalenol, decahydro-1,1,4a,6-tetramethyl-5-[2-[(1*S*,4a*R*,6*S*,8a*R*)-1,4,4a,5,6,7,8,8a-octahydro-6-hydroxy-2,5,5,8a-tetramethyl-1-naphthalenyl]ethyl]-, (2*S*,4a*R*,5*S*,6*S*,8a*R*)-

3 References | 0 Reactions | 0 Suppliers

Key Physical Properties	Value	Condition
Molecular Weight	444.73	-
Boiling Point (Predicted)	522.2±50.0 °C	Press: 760 Torr
Density (Predicted)	0.962±0.06 g/cm^3	Temp: 20 °C; Press: 760 Torr
pKa (Predicted)	15.18±0.70	Most Acidic Temp: 25 °C

Figure S39: Scifinder reports with similarity score 98 to compounds **1**.

3 Similarity Score: **95**

6049-24-7

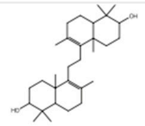

$C_{30}H_{50}O_2$

(2*S*,2'*S*,4a*S*,4'a*S*,8a*R*,8'a*R*)-5,5'-(1,2-Ethanediyl)bis[1,2,3,4,4a,7,8,8a-octahydro-1,1,4a,6-tetramethyl-2-naphthalenol]

8 References | 4 Reactions | 1 Supplier

Key Physical Properties	Value	Condition
Molecular Weight	442.72	-
Boiling Point (Predicted)	527.4±50.0 °C	Press: 760 Torr
Density (Predicted)	0.985±0.06 g/cm^3	Temp: 20 °C; Press: 760 Torr
pKa (Predicted)	14.86±0.70	Most Acidic Temp: 25 °C

Experimental Properties | Spectra

Figure S40: Scifinder reports with similarity score 95 to compounds **1**

CAS SciFinder

Task History

Initiating Search

October 11, 2022, 10:57AM

Substances:

Filtered By:

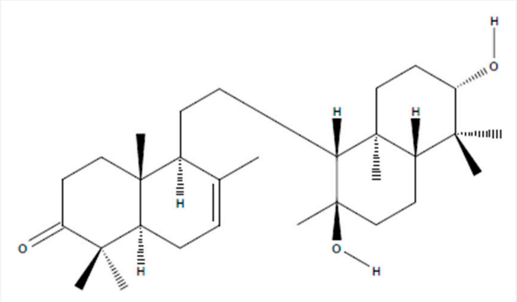

Structure Match: **As Drawn**

Search Tasks

Task	Search Type	View
Exported: Returned Substance Results + Filters	**Substances**	View Results

CAS SciFinder

Substances (0)

View in SciFinder^n

We couldn't find any results. Please update your search query and try again.

Substances with (0) results

Copyright © 2022 American Chemical Society (ACS). All Rights Reserved.
Internal use only. Redistribution is subject to the terms of your SciFinder^n License Agreement and CAS information Use Policies.

Figure S41: Scifinder reports of compounds **2**.

CAS SciFinder^{n}

Substances (12)

View in SciFinder^{n}

1	Similarity Score: **99**

1246091-10-0

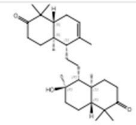

Absolute stereochemistry shown,
Rotation (-)

$C_{30}H_{48}O_3$

(4a*S*,5*R*,6*R*,8a*R*)-Octahydro-6-hydroxy-1,1,4a,6-tetramethyl-5-[2-[(1*S*,4a*R*,8a*R*)-1,4,4a,5,6,7,8,8a-octahydro-2,5,5,8a-tetramethyl-6-oxo-1-naphthalenyl]ethyl]-2(1*H*)-naphthalenone

2 References | 0 Reactions | 0 Suppliers

Key Physical Properties	Value	Condition
Molecular Weight	456.70	-
Boiling Point (Predicted)	544.9±50.0 °C	Press: 760 Torr
Density (Predicted)	1.000±0.06 g/cm^3	Temp: 20 °C; Press: 760 Torr
pKa (Predicted)	15.12±0.70	Most Acidic Temp: 25 °C

Experimental Properties | Spectra

2	Similarity Score: **99**

2649754-72-1

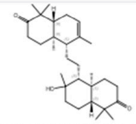

Relative stereochemistry shown

$C_{30}H_{48}O_3$

0 References | 0 Reactions | 1 Supplier

Key Physical Properties	Value	Condition
Molecular Weight	456.70	-
Boiling Point (Predicted)	544.9±50.0 °C	Press: 760 Torr
Density (Predicted)	1.000±0.06 g/cm^3	Temp: 20 °C; Press: 760 Torr
pKa (Predicted)	15.12±0.70	Most Acidic Temp: 25 °C

Figure S42: Scifinder reports with similarity score 99 to compounds **2**

CAS SciFinder^{n}

Substances (21)

View in SciFinder^{n}

1 — Similarity Score: **95**

1613602-59-7

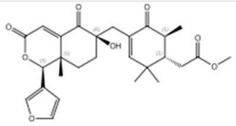

Available stereochemistry shown, Rotation (+)

$C_{27}H_{32}O_8$

rel-(+)-Methyl (1*S*,6*S*)-4-[[(1*R*,6*S*,8a*R*)-1-(3-furanyl)-3,5,6,7,8,8a-hexahydro-6-hydroxy-8a-methyl-3,5-dioxo-1*H*-2-benzopyran-6-yl]methyl]-2,2,6-trimethyl-5-oxo-3-cyclohexene-1-acetate

1 Reference | 0 Reactions | 0 Suppliers

Key Physical Properties	Value	Condition
Molecular Weight	484.54	-
Boiling Point (Predicted)	625.5±55.0 °C	Press: 760 Torr
Density (Predicted)	1.28±0.1 g/cm^3	Temp: 20 °C; Press: 760 Torr
pKa (Predicted)	11.86±0.60	Most Acidic Temp: 25 °C

2 — Similarity Score: **95**

1621959-55-4

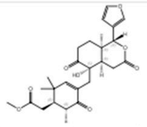

Absolute stereochemistry shown, Rotation (+)

$C_{27}H_{34}O_8$

Methyl (1*R*,6*R*)-4-[[(1*R*,4a*R*,5*S*,8a*R*)-1-(3-furanyl)octahydro-5-hydroxy-8a-methyl-3,6-dioxo-1*H*-2-benzopyran-5-yl]methyl]-2,2,6-trimethyl-5-oxo-3-cyclohexene-1-acetate

1 Reference | 0 Reactions | 0 Suppliers

Key Physical Properties	Value	Condition
Molecular Weight	486.56	-
Boiling Point (Predicted)	648.2±55.0 °C	Press: 760 Torr
Density (Predicted)	1.206±0.06 g/cm^3	Temp: 20 °C; Press: 760 Torr
pKa (Predicted)	11.58±0.60	Most Acidic Temp: 25 °C

Figure S43: Scifinder reports with similarity score 95 to compounds **2**

CAS SciFinder^n Task History

Initiating Search

October 11, 2022, 10:58AM

Substances:

Filtered By:

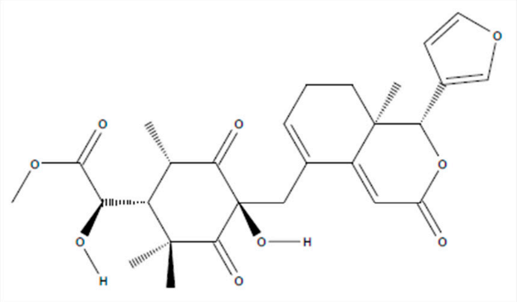

Structure Match: **As Drawn**

Search Tasks

Task	Search Type	View
Exported: Returned Substance Results + Filters	**Substances**	View Results

CAS SciFinder^n

Substances (0)

View in SciFinder^n

We couldn't find any results. Please update your search query and try again.

Substances with (0) results

Copyright © 2022 American Chemical Society (ACS). All Rights Reserved.
Internal use only. Redistribution is subject to the terms of your SciFinder^n License Agreement and CAS information Use Policies.

Figure S44 Scifinder reports of compounds **3**.

3			Similarity Score: **95**

2580943-28-6

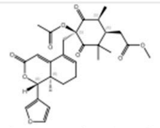

$C_{29}H_{34}O_9$

Cyclohexaneacetic acid, 4-(acetyloxy)-4-[[(1*R*,8a*R*)-1-(3-furanyl)-3,7,8,8a-tetrahydro-8a-methyl-3-oxo-1*H*-2-benzopyran-5-yl]methyl]-2,2,6-trimethyl-3,5-dioxo-, methyl ester, (1*R*,4*S*,6*S*)-

1 Reference | 0 Reactions | 0 Suppliers

Key Physical Properties	Value	Condition
Molecular Weight	526.58	-
Boiling Point (Predicted)	661.5±55.0 °C	Press: 760 Torr
Density (Predicted)	1.26±0.1 g/cm^3	Temp: 20 °C; Press: 760 Torr

Figure S45: Scifinder reports with similarity score 95 to compounds **3**

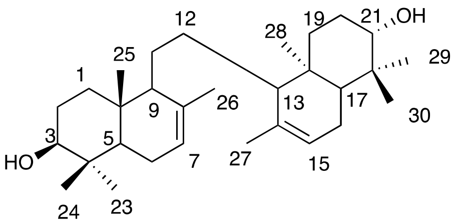

1

Table S1: NMR data comparison of compound **1** with 3-hydroxy-8,14-secogammacera-7,14-dien-21-one [21].

No.	**1**		3-hydroxy-8,14-secogammacera-7,14-dien-21-one [21]	
	^{13}C NMR δc	^{1}H NMR δ_H (Integral, mult, *J*=Hz)	^{13}C NMR δc	^{1}H NMR δ_H (Integral, mult, *J*=Hz)
1	30.1	1.25, 1.45 (each 1H, m)	37.5	1.14, 1.86 (each 1H, m)
2	27.6	1.62, 1.66 (each 1H, m)	27.4	1.65 (2H, m)
3	79.3	3.26 (1H, dd, 11.0, 4.0)	79.1	3.29 (1H, m)
4	38.9	-	38.7	-
5	49.8	1.20 (1H, m)	51.5	1.62 (1H, m)
6	23.7	1.97 (2H, m)	29.8	1.35, 1.50 (each 1H, m)
7	122.1	5.39 (1H, brs)	121.7	5.40 (1H, m)
8	135.4	-	135.4	-
9	56.3	1.59 (1H, m)	55.3	1.66 (1H, m)
10	36.7	-	36.5	-
11	37.6	1.17, 1.88 (each 1H, m)	24.1	1.99, 2.10 (each 1H, m)
12	37.6	1.17, 1.88 (each 1H, m)	23.5	1.98, 2.10 (each 1H, m)
13	56.3	1.59 (1H, m)	56.0	1.59 (1H, m)
14	135.4	-	134.9	-
15	122.1	5.39 (1H, brs)	122.3	5.40 (1H, m)
16	23.7	1.97 (2H, m)	29.9	1.35, 1.50 (each 1H, m)
17	49.8	1.20 (1H, m)	49.6	1.20 (1H, m)
18	36.7	-	36.5	-
19	30.1	1.25, 1.45 (each 1H, m)	38.4	1.51, 2.15 (each 1H, m)
20	27.6	1.62, 1.66 (each 1H, m)	34.7	2.29, 2.77 (each 1H, m)
21	79.3	3.26 (1H, dd, 11.0, 4.0)	217.0	-
22	38.9	-	47.5	-
23	15.3	0.85 (3H, s)	15.1	0.87 (3H, s)
24	28.1	0.98 (3H, s)	27.9	1.00 (3H, s)
25	13.8	0.74 (3H, s)	13.3	0.99 (3H, s)
26	22.6	1.70 (3H, s)	22.3	1.75 (3H, s)
27	22.6	1.70 (3H, s)	22.4	1.72 (3H, s)
28	13.8	0.74 (3H, s)	13.6	0.76 (3H, s)
29	15.3	0.85 (3H, s)	22.1	1.12 (3H, s)
30	28.1	0.98 (3H, s)	25.0	1.08 (3H, s)

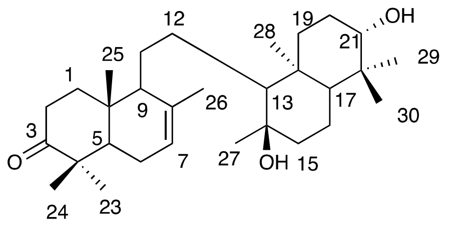

2

Table S2: NMR data comparison of compound **2** with kokosanolide B [14].

No.	**2**		kokosanolide B [14]	
	^{13}C NMR δc	^{1}H NMR δ_H (Integral, mult, *J*=Hz)	^{13}C NMR δc	^{1}H NMR δ_H (Integral, mult, *J*=Hz)
1	38.3	1.44, 2.10 (each 1H, m)	38.5	1.91, 2.08 (each 1H, m)
2	34.9	2.26, 2.72 (each 1H, m)	34.8	2.23, 2.41 (each 1H, m)
3	217.4	-	216.9	-
4	47.6	-	47.6	-
5	51.6	1.57 (1H, m)	51.6	1.57 (1H, m)
6	28.8	1.40, 1.84 (each 1H, m)	28.9	1.12, 2.56 (each 1H, m)
7	121.6	5.40 (1H, brs)	121.7	5.41 (1H, m)
8	135.9	-	135.3	-
9	55.4	1.59 (1H, m)	55.5	1.59 (1H, m)
10	38.7	-	36.6	-
11	20.4	1.32, 1.66 (each 1H, m)	21.5	1.61, 2.41 (each 1H, m)
12	24.2	1.22, 1.88 (each 1H, m)	21.5	1.62, 1.76 (each 1H, m)
13	62.6	0.99 (1H, m)	61.8	1.12 (1H, m)
14	74.3	-	74.0	-
15	44.8	1.36, 1.86 (each 1H, m)	44.2	1.46, 2.23 (each 1H, m)
16	31.5	1.36, 1.40 (each 1H, m)	31.4	1.51, 1.84 (each 1H, m)
17	55.2	0.88 (1H, m)	55.2	1.42 (1H, m)
18	36.6	-	36.6	-
19	38.0	1.10, 1.65 (each 1H, m)	38.4	1.78, 2.10 (each 1H, m)
20	27.3	1.59, 1.68 (each 1H, m)	34.1	2.26, 2.73 (each 1H, m)
21	78.9	3.23 (1H, dd, 11.5; 4.0)	217.1	-
22	39.0	-	47.6	-
23	22.3	1.09 (3H, s)	22.3	1.08 (3H, s)
24	25.1	1.04 (3H, s)	25.1	1.04 (3H, s)
25	13.4	0.96 (3H, s)	13.4	0.96 (3H, s)
26	22.3	1.76 (3H, s)	22.3	1.77 (3H, s)
27	24.3	1.15 (3H, s)	24.2	1.21 (3H, s)
28	15.8	0.77 (3H, s)	15.1	0.93 (3H, s)
29	28.3	0.99 (3H, s)	21.4	1.02 (3H, s)
30	15.5	0.75 (3H, s)	26.5	1.09 (3H, s)

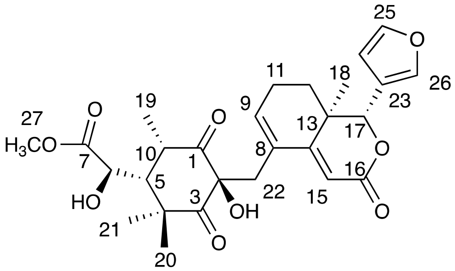

Table S3: NMR data comparison of compound **3** with kokosanolide A [14].

No.	**3**		kokosanolide A [14]	
	^{13}C NMR δc	^{1}H NMR δ$_H$ (Integral, mult, *J*=Hz)	^{13}C NMR δc	^{1}H NMR δ$_H$ (Integral, mult, *J*=Hz)
1	211.5	-	108.4	-
2	80.4	-	76.1	-
3	208.1	-	208.6	-
4	47.3	-	48.4	-
5	53.0	2.18 (1H, dd, 1.5, 1.0)	56.4	2.27 (1H, dd, 4.0, 7.0)
6	73.8	4.32 (1H, d, 1.0)	77.4	4.81 (1H, d, 4.0)
7	174.1	-	172.4	-
8	126.7	-	34.6	2.64 (1H, dd, 5.3, 6.5)
9	141.5	6.39 (1H, t, 1.0)	69.5	4.39 (1H, m)
10	43.2	3.06 (1H, m)	37.3	3.33 (1H, q, 8.0)
11	22.5	2.30 (2H, m)	25.3	1.79, 1.91 (each 1H, m)
12	29.7	1.39 (2H, m)	27.8	1.26, 1.74 (each 1H, m)
13	37.7	-	39.6	-
14	159.7	-	168.6	-
15	110.4	5.97 (1H, m)	116.7	6.28 (1H, s)
16	166.3	-	165.4	-
17	80.8	5.02 (1H, s)	81.5	5.17 (1H, s)
18	15.9	0.95 (3H, s)	18.2	1.07 (3H, s)
19	16.0	1.27 (3H, d, 6.4)	12.2	1.13 (3H, d, 8)
20	26.5	0.91 (3H, s)	23.7	0.98 (3H, s)
21	20.2	1.07 (3H, s)	30.1	1.37 (3H, s)
22	36.1	2.86, 3.19 (each 1H, s)	25.4	2.46 (1H, dd, 7.5, 5.3)<br>2.77 (1H, dd, 7.4, 4.7)
23	120.0	-	120.5	-
24	110.2	6.41 (1H, d, 2.0)	110.8	6.43 (1H, d, 4.4)
25	143.1	7.40 (1H, d, 2.0)	143.5	7.40 (1H, d, 4.4)
26	141.1	7.44 (1H, s)	141.9	7.47 (1H, s)
27	53.0	3.80 (3H, s)	52.6	3.67 (3H, s)

Table S4: Results of cytotoxic activity of **1** against MCF-7 cell line.

Concentration of sample (µg/mL)	% Inhibition		
	1	2	3
500	99.2414	98.4165	100.1733
100	98.7816	99.7180	99.1832
10	16.7126	27.2668	1.1634
1	16.7126	25.5315	13.7871

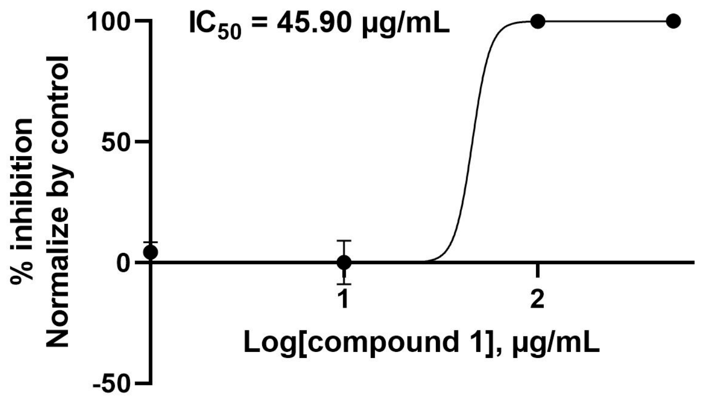

Table S5: Results of cytotoxic activity of **2** against MCF-7 cell line.

Concentration of sample (µg/mL)	% Inhibition		
	1	2	3
500	99.1453	101.9066	99.9343
100	13.9382	9.2045	20.6443
50	9.2045	10.5851	10.3879
10	11.1769	6.2459	13.1492
1	1.9066	0.8547	7.4293

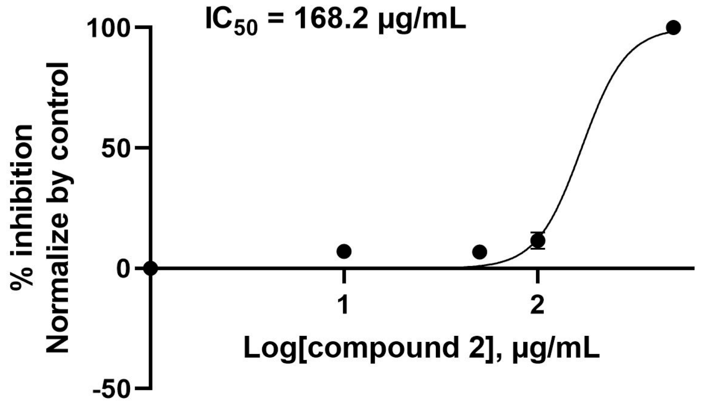

Table S6: Results of cytotoxic activity of **3** against MCF-7 cell line.

Concentration of sample	% inhibition

(μg/mL)	1	2	3
500	99.06617	99.22625	99.62647
250	75.21345	70.97118	68.89007
100	60.24546	55.68303	55.84312
10	45.91782	44.39701	52.48132
1	36.23266	40.47492	37.75347

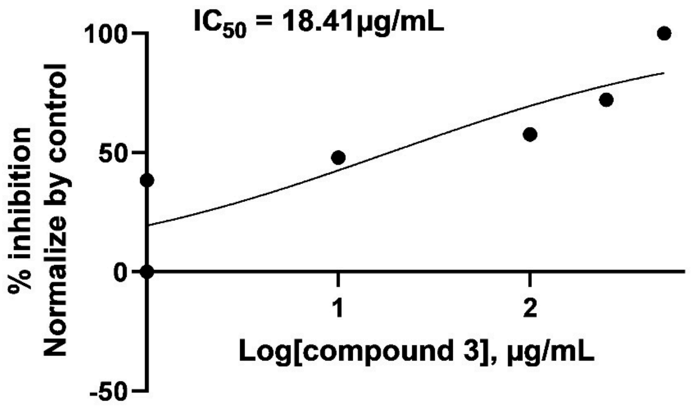

References

[14] Mayanti, T.; Tjokronegoro, R.; Supratman, U.; Mukhtar, M.R.; Awang, K.; Hadi, A.H.A. Antifeedant triterpenoids from the seeds and bark of *Lansium domesticum* Corr. cv *kokossan* (Meliaceae), Molecules. **2011,** 16, 2785-2795.

[21] Zulfikar; Putri, N.K; Fajriah, S.; Yusuf, M.; Maharani, R.; Al Anshori, J.; Supratman U.; Mayanti, T. 3-hydroxy-8,14-secogammacera-7,14-dien-21-one: A new onoceranoid triterpenes from *Lansium domesticum* Corr. cv *kokossan*, Molbank **2020**, 4, M1157.
